# Rapid determination of anti-tuberculosis drug resistance from whole-genome sequences

**DOI:** 10.1186/s13073-015-0164-0

**Published:** 2015-05-27

**Authors:** Francesc Coll, Ruth McNerney, Mark D Preston, José Afonso Guerra-Assunção, Andrew Warry, Grant Hill-Cawthorne, Kim Mallard, Mridul Nair, Anabela Miranda, Adriana Alves, João Perdigão, Miguel Viveiros, Isabel Portugal, Zahra Hasan, Rumina Hasan, Judith R Glynn, Nigel Martin, Arnab Pain, Taane G Clark

**Affiliations:** Faculty of Infectious and Tropical Diseases, London School of Hygiene & Tropical Medicine, Keppel Street, London, WC1E 7HT UK; Advanced Data Analysis Centre, University of Nottingham, Wollaton Road, Nottingham, NG8 1BB UK; Biological and Environmental Sciences and Engineering Division, King Abdullah University of Science and Technology (KAUST), Thuwal, Kingdom of Saudi Arabia; Sydney Emerging Infections and Biosecurity Institute and School of Public Health, University of Sydney, Sydney, Australia; Tuberculosis Laboratory, Instituto Nacional de Saude Dr. Ricardo Jorge, Porto, Portugal; Centro de Patogénese Molecular, Faculdade de Farmácia da Universidade de Lisboa, Lisbon, Portugal; Grupo de Micobactérias, Unidade de Microbiologia Médica, Instituto de Higiene e Medicina Tropical, Universidade Nova de Lisboa, Lisbon, Portugal; Department of Pathology & Microbiology, Aga Khan University Hospital, Karachi, Pakistan; Karonga Prevention Study, Chilumba, Malawi; Department of Computer Science, Birkbeck College, University of London, Malet Street, London, WC1E 7HX UK

## Abstract

*Mycobacterium tuberculosis* drug resistance (DR) challenges effective tuberculosis disease control. Current molecular tests examine limited numbers of mutations, and although whole genome sequencing approaches could fully characterise DR, data complexity has restricted their clinical application. A library (1,325 mutations) predictive of DR for 15 anti-tuberculosis drugs was compiled and validated for 11 of them using genomic-phenotypic data from 792 strains. A rapid online ‘TB-Profiler’ tool was developed to report DR and strain-type profiles directly from raw sequences. Using our DR mutation library, *in silico* diagnostic accuracy was superior to some commercial diagnostics and alternative databases. The library will facilitate sequence-based drug-susceptibility testing.

**Electronic supplementary material:**

The online version of this article (doi:10.1186/s13073-015-0164-0) contains supplementary material, which is available to authorized users.

## Background

Resistance has been reported to all drugs used to treat tuberculosis (TB) [1]. Increased resistance is associated with decreased patient survival and is a substantial threat to disease control. The World Health Organization (WHO) classifies tuberculosis resistant to isoniazid and rifampicin as multi drug-resistant (MDR-TB), when a switch to second line treatment is advised. Resistance to additional drugs further compromises treatment success [2]. MDR-TB strains that have developed resistance to the fluoroquinolones and aminoglycosides are classed as extensively drug resistant (XDR-TB). The term totally drug resistant (TDR-TB) has been used to describe strains found resistant to all available drugs, but there is not yet an agreed definition of TDR-TB [1]. Treatment of drug resistant disease is prolonged and expensive, and outcomes are poor [2,3]. Treatment involves drugs of heightened toxicity and adverse reactions are common and may be severe and irreversible [4,5]. Poor tolerance leads to reduced compliance, which in turn reduces cure rates and can result in amplification of resistance [6].

Early detection is crucial for access to effective treatment and prevention of onward transmission. Knowledge of the full drug susceptibility profile would enable tailored treatment to improve efficacy and reduce exposure to ineffective toxic drugs. Current testing for resistance to most anti-TB drugs involves isolation and culture of the bacteria followed by exposure to the drug, a process that takes weeks or months and requires high levels of microbiological safety. The primary cause of resistance in *M. tuberculosis* is the accumulation of point mutations and insertions and deletions (indels) in genes coding for drug-targets or -converting enzymes [7]. Rapid molecular assays that test directly from sputum are available for some key drugs. In 2013 the Xpert MTB/RIF (Cepheid, Inc., Sunnyvale, CA, USA), was granted US FDA approval for detecting resistance to rifampicin, conditional on confirmatory testing [8]. This easy to use semi-automated PCR-based test has also been endorsed by WHO, as have Line Probe Assays (LPA) for resistance to rifampicin and isoniazid, where, following amplification of bacterial DNA, samples are interrogated with a panel of oligonucleotide probes [9]. LPA to detect resistance to other drugs, including fluoroquinolones and aminoglycosides, have also been developed [10], but have yet to be endorsed by WHO. Though undoubtedly useful, both technologies are limited in the number of loci they examine and they lack capacity to differentiate silent mutations from those that effect drug efficacy, leading to false positive results [11-13]. Whole genome sequencing has the potential to overcome such problems and extend rapid testing to the full range of anti-TB drugs and has been applied in a clinical setting. Bench top analysers have been developed capable of sequencing a bacterial genome in a few hours and costs have been greatly reduced with the introduction of high throughput technology. Sequencing already assists patient management for a number of conditions such as HIV for which Sanger sequencing is performed to determine viral tropism and drug susceptibility [14]. Recent reports of sequencing *M. tuberculosis* from sputum from suspected XDR-TB patients suggests it has a role in the management of TB [15-17]. However, data analysis remains a bottleneck, requiring specialist expertise not readily available in clinical laboratories. To address this issue and progress sequencing towards real time management of patients we have compiled an exhaustive library of 1,325 drug resistance markers and developed an online tool that rapidly analyses raw sequence data and predicts resistance. We present accuracy data comparing *in silico* whole genome analysis for resistance to 11 anti-TB drugs, to conventional drug susceptibility testing (DST). To further assess potential benefits of the whole genome approach we compared our curated mutation database to two others (*TBDreaMDB* and *MUBII-TB-DB*), as well as those used in three commercial molecular tests, the Xpert MTB/RIF (Cepheid, Inc., Sunnyvale, CA, USA), and the MTBDRplus and MTBDRsl (Hain Life Science, Germany). In particular, *in silico* versions of the three commercial molecular tests were implemented.

## Methods

### Mutation library

**Figure 1:**
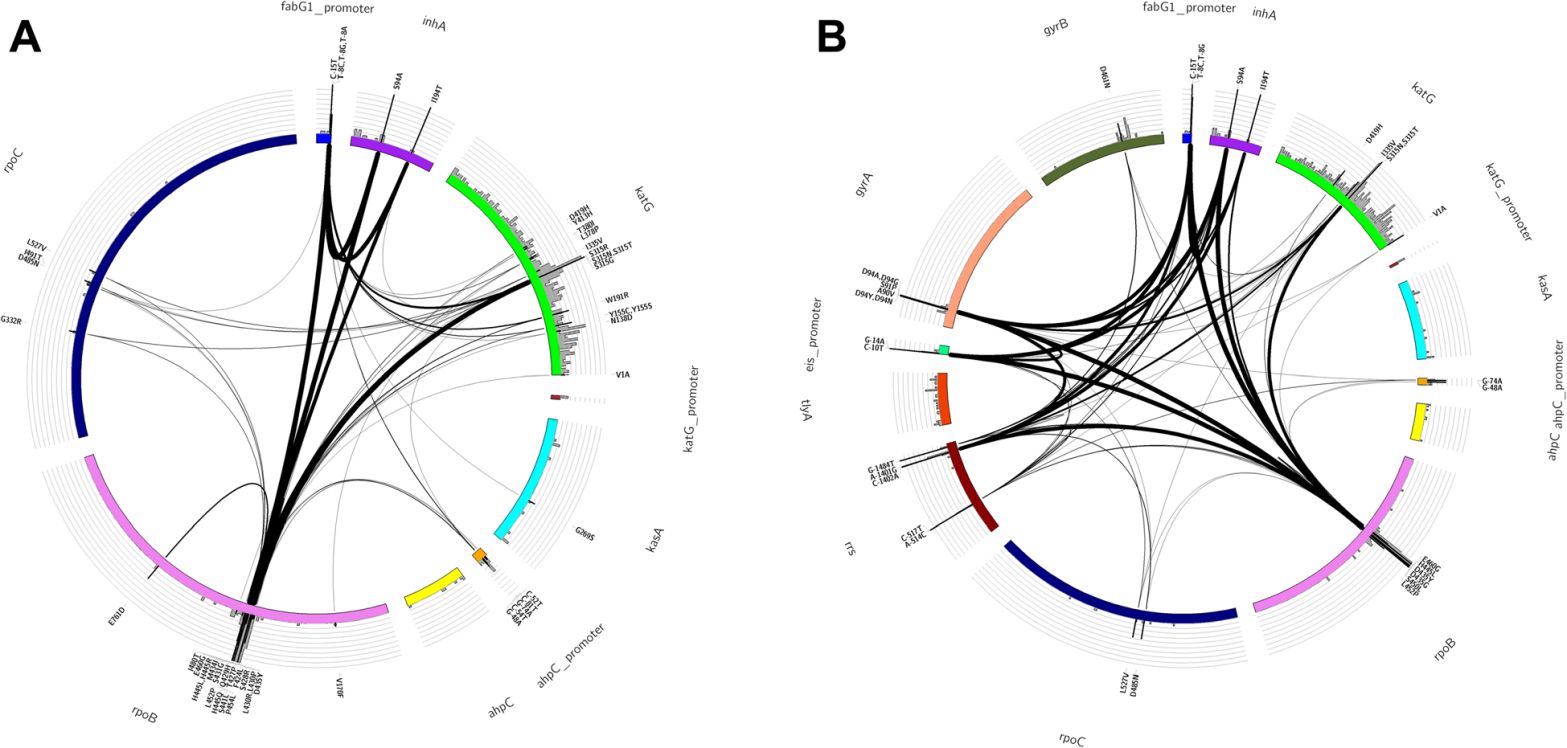
Polymorphism in the curated library used for predicting multi-drug resistant TB (MDR-TB) and extensive-drug resistant TB (XDR-TB). **(A)** Polymorphisms associated with MDR-TB. **(B)** Polymorphisms associated with XDR-TB. Colour-coded bars in the Circos plot represent genes described to be involved in drug resistance (from Table 1). On top of each of these bars a grey histogram shows the mutation density (calculated as the number of polymorphic sites within windows of 20 bp) derived from the curated list of DR-associated mutations. These grey areas highlight the presence of DR-associated regions in candidate genes, which in some cases span the whole gene (for example, *katG*) or are confined to a certain region of the gene (for example, *rpoB*). Vertical black lines indicate the frequency of mutations (that is, the number of times the mutation has been observed) in phenotypically resistance isolates. Internal black lines show co-occurring mutations both within and between genes. The thickness of these lines is proportional to the frequency of the mutations appearing together.

Following review of available data, a library of mutations predictive of drug resistance was compiled. First, mutations from two publically available web-based tools *TBDreaMDB* [18] and *MUBII-TB-DB* [19] were extracted. Second, phylogenetic SNPs at drug resistance loci were removed (see Additional file 1: Table S2 for the full list), as they have been historically misclassified as drug resistance markers [20,21]. And third, recent literature was consulted to include mutations and loci not described in *TBDreaMDB* and *MUBII-TB-DB*. (See Additional file 1: Table S1 for a list of source materials). Drugs included were amikacin (AMK), capreomycin (CAP), ethambutol (EMB), ethionamide (ETH), isoniazid (INH), kanamycin (KAN), moxifloxacin (MOX), ofloxacin (OFX), pyrazinamide (PZA), rifampicin (RMP), streptomycin (STR), para-aminosalicylic acid (PAS), linezolid (LZD), clofazimine (CFZ) and bedaquiline (BDQ). As presented in Table 1, the library comprised 1,325 polymorphisms (SNPs and indels) at 992 nucleotide positions from 31 loci, six promoters and 25 coding regions (see [22] for full list). In addition to examining individual drugs we considered the cumulative loci for MDR- and XDR-TB. Circos software [23] was used to construct circular genomic region variation maps. Polymorphisms associated with MDR- and XDR-TB are shown in Figure 1 (See Additional file 1: Figure S1 for full details).

**Table 1:** **Summary of mutations included in the curated whole genome drug resistance library**

**Drug**	**Loci**	**No. variable sites**	**SNPs**	**Indels**
INH	*katG*	241	286	25
	*katG* promoter	3	3	0
	*inhA*	12	15	0
	*inhA* promoter	9	11	0
	*ahpC*	8	8	0
	*ahpC* promoter	13	14	0
	*kasA*	8	11	0
RMP	*rpoB*	89	135	19
	*rpoC*	8	8	0
EMB	*embB*	123	153	1
	*embA*	5	5	0
	*embA* promoter	3	3	0
	*embC*	25	26	0
	*embR*	22	24	0
STR	*rrs*	21	25	0
	*rpsL*	14	19	0
PZA	*pncA*	215	269	64
	*pncA* promoter	4	6	0
	*rpsA*	3	4	0
	*panD*	9	11	1
ETH	*ethA*	33	29	5
	*ethR*	3	4	0
	*inhA* promoter	3	3	0
	*inhA*	3	3	0
FLQs	*gyrA*	15	22	0
	*gyrB*	22	29	0
AMK	*rrs*	8	9	0
CAP	*rrs*	3	4	0
	*tlyA*	26	18	10
KAN	*rrs*	3	4	0
	*eis* promoter	9	10	0
PAS	*thyA*	23	17	5
	*folC*	16	19	0
	*ribB*	1	1	0
LZD	*rrl*	2	2	0
	*rplC*	1	1	0
BDQ CFZ	*Rv0678*	7	5	2

AMK, amikacin; BDQ, bedaquiline; CAP, capreomycin; CFZ, clofazimine; EMB, ethabutol; ETH, ethionamide; FLQs, fluoroquinolones; INH, isoniazid; KAN, kanamycin; LZD, linezolid; PAS, para-aminosalycylic acid; PZ, pyrazinamide; RMP, rifampicin; STR, streptomycin.

### Sequence data and drug susceptibility testing

The precision of the curated library for predicting resistance was assessed through analysis of new and published sequence data. *In silico* inferred resistance phenotypes were compared to phenotypes derived from conventional culture-based methods with the exception of PAS, LZD, CFZ and BDQ, for which insufficient phenotypic DST were available for comparison. Six geographically distinct datasets were used: China (n = 161) [24], Karachi, Pakistan (n = 42) [25], Karonga District, Malawi (n = 337) [26], Lisbon and Porto, Portugal (n = 208) [27], Samara, Russia [28] (n = 21) and Vancouver, Canada (n = 19) [29] (See Additional file 1: Table S3). Strains used in the study are a convenience sample and do not necessarily reflect the population at the site of collection. All collections had Illumina raw sequencing data (minimum read length 50 bp) and drug susceptibility data from recognised testing protocols [30]. Where conventional susceptibility data was not available, samples were excluded from analysis for that drug. Sensitivity, specificity and diagnostic accuracy (area under the receiver operating characteristic curve) were estimated using the phenotypic drug susceptibility test result as the reference standard [31]. *P* values and confidence intervals were determined using binomial distribution approximations.

### Rapid mutation detection and the TB Profiler Online tool

To rapidly characterise mutations from whole genome sequence files (*fastq* format), we map raw sequences to a modified version of the H37Rv reference genome (Genbank accession number: NC_000962.3) using the *Snap* algorithm [32], and call SNPs and indels using *samtool/vcf* tools of high quality (Q30, 1 error per 1,000 bp) as previously described [21,33]. The modified reference genome consists of the genes and flanking regional sequences containing the 1,325 drug resistance mutations in the curated list (Table 1) and selected lineage specific mutations [21]. All high quality SNPs and indels identified from the alignments are compared to the curated list to determine known and novel polymorphism. Algorithmic results obtained were compared to standard SNP calling procedures using the full reference genome [21]. The online *TB Profiler* tool [34] was developed in Perl/PHP. It inputs raw sequence data (*fastq* format), identifies drug resistance and lineage specific mutations, and displays related outputs (see screenshots in Additional file 1: Figure S2). A Perl script was used to implement the *Snap* software and *samtool/vcf* based bioinformatic pipeline. The script is available from the corresponding author.

#### Comparison with existing tools

**Figure 2:**
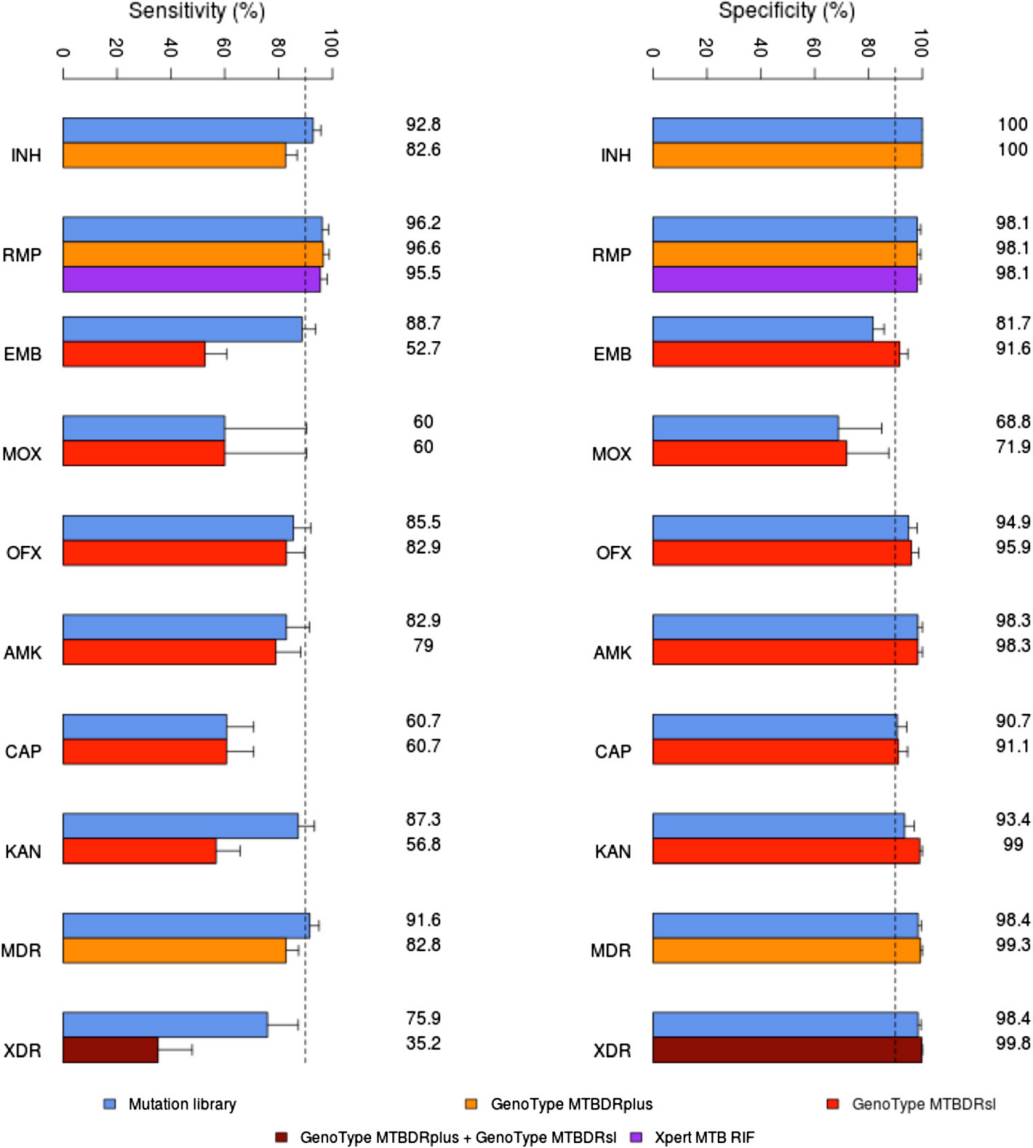
Inferred analytical accuracies of the whole genome mutation library and three commercial molecular tests for resistance. *In silico* analysis of published sequence data using mutation libraries derived from XpertMTB/RIF (Cepheid Inc., USA) (purple), MTBDRsl (red) and MTBDRplus (orange) (Hain Life Sciences, Germany), and the curated whole genome library (blue). For each library *in silico* inferred resistance phenotypes were compared to reported phenotypes obtained from conventional drug susceptibility testing. Errors bars correspond to 95% confidence intervals. Abbreviations: AMK, amikacin; CAP, capreomycin; EMB, ethambutol; ETH, ethionamide; INH, Isoniazid; KAN, kanamycin; MDR, multi-drug resistance; MOX, moxifloxacin; OFX, ofloxacin; PZA, pyrazinamide; RMP, rifampicin; STR, streptomycin; XDR, extensive drug resistance.

To examine the potential analytical advantage of whole genome sequencing comparison was made with three commercial tests: (1) the Xpert MTB/RIF (Cepheid Inc., USA) which targets the *rpo*B gene for RMP resistance; (2) the LPA MTBDRplus for MDR-TB (Hain Lifescience, Germany) which targets *rpo*B, *kat*G and *inh*A for resistance to RMP and INH; and (3) the LPA MTBDRsl (Hain Lifescience, Germany) which targets *gyr*A, *rrs* and *emb*B for resistance to the fluoroquinolones (FLQ), aminoglycosides and ethambutol, respectively. *In silico* versions were developed based on the polymorphisms used by these assays and their performance compared to the whole genome mutation library. In particular, *in silico* analysis of the six datasets was performed and analytical sensitivities and specificities of the inferred resistance relative to the reported phenotype were compared (Figure 2, Additional file 1: Figures S3 and S4). *KvarQ* [35], a new tool that directly scans *fastq* files of bacterial genome sequences for known genetic polymorphisms, was run across all 792 samples using the MTBC test suite and default parameters. Sensitivity and specificity achieved by this method using phenotypic DST results as the reference standard were calculated.

## Results

### A mutation library

Following review of available data (See Additional file 1: Table S1 for a list of source materials), a library comprising 1,325 polymorphisms (single nucleotide polymorphisms (SNPs) and indels) at 992 nucleotide positions from 31 loci, six promoters and 25 coding regions was established. This library covered the anti-TB drugs: EMB, ETH, INH, PZA, RMP, STR and the second line drugs used to treat MDR-TB AMK, CAP, KAN, MOX and OFX. Mutations associated with resistance to PAS, LZD, CFZ and BDQ were also compiled but were not included in the analysis given lack of available phenotypic DST results. In addition to examining individual drugs we considered the cumulative loci for MDR- and XDR-TB. Polymorphisms associated with MDR- and XDR-TB are shown in Figure 1 (see Additional file 1: Figure S1 for full details).

### Validation of the mutation library

The mutation library was validated using new and publically available sequence and phenotypic data from 792 isolates, from six countries (Canada, China, Malawi, Pakistan, Portugal and Russia; see Additional file 1: Table S3). Of the 792 isolates, 365 (46%) were phenotypically resistant to at least one drug, 262 (33%) were MDR-TB, 54 (6.8%) XDR-TB and 426 (54%) were susceptible to all drugs tested. *In silico* genotyping [36] revealed the major modern *M. tuberculosis* lineages were represented, including Lineage 1 (East African Indian spoligotype family: 68, 8.6%), Lineage 2 (Beijing spoligotype: 182, 23%), Lineage 3 (Central Asian: 86, 10.9%) and Lineage 4 (456 isolates, 57.5% including 298 LAM, 35 X, 97 T, 4S, 18 H and 4 other spoligotypes). *In silico* inferred resistance from whole genome sequence data was compared to the reported resistance phenotype from conventional culture-based susceptibility testing. Results are summarised in Table 2. Sensitivity and specificity varied by drug, and with the geographic origin (Additional file 1: Figure S4). For the drugs that contribute to MDR-TB correlation of mutation analysis with the reported phenotype was high. Mutations predictive of resistance were found in 96.0% and 92.8% of samples resistant to RMP and INH, respectively. Of 22 phenotypically INH resistant samples not detected by mutation analysis, 14 were from China. Further analysis revealed seven had mutations in known candidate loci (*katG* and *ahpC* promoter) not previously reported (Additional file 1: Table S4). No additional cases of INH resistance were suggested by the genome analysis. However, 10 isolates reported as susceptible to RMP by conventional testing had mutations predictive of resistance, six of which were from Malawi. Correlation was slightly poorer for other first line drugs. For PZA 32 of 110 samples with a resistant phenotype were not recognised by genome analysis, including 18 of 37 samples from Karachi. However, specificity for this drug was high (93%; 95% CI: 90.6 to 97.2). Correlation was also reduced for EMB where 61 of 334 susceptible stains were found to harbour mutations included in the library of resistance polymorphisms (81.7% specificity). For the aminoglycosides used to treat MDR-TB correlation was higher for AMK and KAN than for CAP, where 35 of 89 resistant samples were not detected by the *in silico* genome analysis. Testing for fluoroquinolone resistance was less commonly reported and data for OFX was restricted to 313 samples from two studies (China and Portugal). Mutations were not identified in 17 resistant samples (85.5% sensitivity) and 10 drug susceptible samples were found to harbour mutations associated with resistance (94.9% specificity). Of 42 samples tested for susceptibility to MOX, 10 were reported as phenotypically resistant, of which six were recognised by the *in silico* mutation analysis.

**Table 2:** **Accuracy of whole genome drug resistance analysis compared to reported resistance phenotype when applied to** ***in silico*** **determination of resistance from raw sequence data.**

**Drug**	**Sample size**	**Resistant (%)**	**Sensitivity (95% CI)**	**Specificity (95% CI)**	**Accuracy (95% CI)**	**China Sen/Spec**	**Pakistan Sen/Spec**	**Malawi Sen/Spec**	**Portugal Sen/Spec**	**Russia Sen/Spec**	**Canada Sen/Spec**
INH	693	305 (44)	92.8 (89.9-95.7)	100 (100–100)	96.8 (95.5-98.1)	88.0/100	100/100	92.6/100	94.6/100	100/100	-/100
RMP	694	264 (38)	96.2 (93.9-98.5)	98.1 (96.8-99.4)	97.4 (96.2-98.5)	95.7/97.7	97.3/100	100/98.2	96.9/100	90.9/90.0	-/100
EMB	484	150 (31)	88.7 (83.6-93.8)	81.7 (77.6-85.8)	83.9 (80.6-87.2)	83.6/71.3	100/42.7	100/80	85.7/68.1	100/80.0	-/100
STR	487	225 (46.2)	87.1 (82.7-91.5)	89.7 (86.0-93.4)	88.5 (85.7-91.3)	86.8/91.0	95.8/44.4	61.5/95.6	86.8/81.5	100/100	-/100
PZA	307	110 (35.8)	70.9 (62.4-79.4)	93.9 (90.6-97.2)	85.7 (81.7-89.6)	NT	51.3/-	66.7/94.8	80.6/100	100/60.0	-/100
ETH	334	155 (46.4)	73.6 (66.7-80.5)	93.3 (89.6-97.0)	84.1 (80.2-88.1)	38.9/97.3	66.7/90.3	NT	84.9/84.6	NT	NT
MOX	42	10 (23.8)	60.0 (29.6-90.4)	68.7 (52.6-84.8)	66.7 (52.4-80.9)	NT	NT	NT	83.3/56.2	25.0/100	NT
OFX	313	117 (37.4)	85.5 (79.1-91.9)	94.9 (91.8 · 98.0)	91.4 (88.3-94.5)	77.8/95.1	-/100	NT	92.1/93.2	NT	NT
AMK	193	76 (39.4)	82.9 (74.4-91.4)	98.3 (96.0-100)	92.2 (88.4-96.0)	NT	86.5/100	NT	79.5/98.2	NT	NT
CAP	358	89 (24.9)	60.7 (50.6-70.8)	90.7 (87.2-94.2)	83.2 (79.4-87.1)	50.0/97.0	85.7/21.7	NT	57.7/98.0	100/91.7	NT
KAN	118	118 (37.3)	87.3 (81.3-93.3)	93.4 (89.9-96.9)	91.1 (88.0-94.3)	71.4/97.0	83.8/-	NT	98.0/88.7	80.0/33.3	NT
MDR	693	262 (37.8)	91.2 (87.8-94.6)	98.4 (97.2-99.6)	95.8 (94.3-97.3)	86.3/100	97.3/100	100/98.2	95.8/100	90.9/90.0	-/100
XDR	601	54 (9)	75.9 (64.5-87.3)	98.4 (97.3-99.5)	96.3 (94.8-97.8)	60.9/99.1	-/100	-/100	96.3/88.9	25.0/100	-/100

AMK, amikacin; CAP, capreomycin; CI, confidence interval; EMB, ethambutol; ETH, ethionamide; INH, isoniazid; KAN, kanamycin; MDR, multi-drug resistance; MOX, moxifloxacin; OFX, ofloxacin; NT, not tested; PZA, pyrazinamide; RMP, rifampicin; Sen, sensitivity; Spec, specificity; STR, streptomycin; XDR, extensive drug resistance.

### Comparison with commercial tests and other drug resistance databases

Having assessed the diagnostic potential of the mutation library, comparison was made with the polymorphisms used in commercially available molecular tests for drug resistance. Results are summarised in Figure 2. There was no significant difference between the mutation library and polymorphisms employed by the Xpert MTB/RIF and the LPA MTBDRplus for detecting resistance to RMP. However, 31 samples had mutations predictive of resistance to INH not covered by the MTBDRplus. The alleles concerned were mainly in the gene encoding catalase-peroxidase enzyme (*katG*) (S315N (n = 9), S315G (n = 1), D419H (n = 1), L378P (n = 1), V1A (n = 1), Y155C (n = 3), W191R (n = 5 and always with C-15T *inhA* promoter), N138D (n = 1, with T-8A *inhA* promoter) and T380I (n = 1; with C-15T *inhA* promoter). There were also six samples with *ahpC* promoter mutations and two samples with *inhA* mutations (S94A and I194T). No resistance mutations were observed in INH susceptible strains suggesting 100% specificity. Overall, when screening for MDR-TB the mutation library offered enhanced accuracy over the line probe mutations (95.8 vs. 93.1%; *P* <0.0004) (Table 2).

Fewer susceptibility data were available for the second line drugs. For each of the fluoroquinolones and aminoglycosides the sensitivity of the mutation library was equal to, or greater than for the mutations employed in the LPA MTBDRsl (Figure 2), although a slight reduction in specificity was observed: MOX (71.9 vs. 68.8%, *P* <0.32), OFX (95.9 vs. 94.9%, *P* <0.083), CAP (91.1 vs. 90.7%, *P* <0.32), KAN (99.0 vs. 93.4%, *P* <0.001) and EMB (86.6 vs. 81.7%, *P* <0.001). Overall when detecting XDR-TB the whole genome analysis offered enhanced accuracy over the line probe assay (96.3 vs. 93.7%; *P* <0.0047) (Table 2).

The mutation library was also found to be more accurate than previously reported databases *TBDreaMDB* and *MUBII-TB-DB* (Additional file 1: Figure S3), because of false positive resistance arising in those databases due to the inclusion of some phylogenetic (but not drug resistance) informative SNPs. An improvement in sensitivity was also achieved for INH, EMB, ETH, PZA and KAN by considering recently discovered drug resistance loci and polymorphisms (Additional file 1: Figure S3).

When compared to *KvarQ* [35] the mutation library achieved higher sensitivity for resistance to isoniazid, pyrazinamide, ofloxacin and amikacin with increases of 5.9%, 8.2%, 3.5% and 7.9%, respectively, without compromising specificity (Additional file 1: Table S5). Higher sensitivity was also achieved for ethambutol (28%), streptomycin (7.1%) and kanamycin (33.1%) but with reductions in specificity (-7.5%, -9.1% and -5.1%, respectively). Sensitivity and specificity values remained the same or very similar for rifampicin and moxifloxacin. *KvarQ* did not predict resistance status for ethionamide and capreomycin.

### Online tool for predicting drug resistance and lineage information from sequenced isolates

Having established a curated list of 1,325 mutations predictive of resistance, we sought to develop a web-based tool to rapidly identify a DST and strain-type profile. Our approach called ‘*TB Profiler*’ ([34], Additional file 1: Figure S2) aligns raw sequencing data to an abridged reference genome covering genomic regions of interest. The alignment is robust to indels and genomic frameshifts, and can be completed in minutes. Detection of *M. tuberculosis* lineage specific markers was also incorporated [21]. In addition to identifying known drug resistance associated mutations, the tool also identifies other mutations in the candidate regions. *TB profiler* processed *fastq* files at a linear rate of 80,000 sequence reads per second. Application to the 792 samples led to the identification of 38 novel mutations (24 non-synonymous SNPs, 9 indels and 5 intergenic SNPs) present in phenotypically resistant strains but absent in susceptible ones (Additional file 1: Table S4). All mutations were confirmed by the alignment of the short reads to the whole H37Rv genome reference sequence using established genome analysis pipelines [21]. The median run-time for the *TB Profiler* was 5 min (range, 2 to 10 min) across samples with depth of coverage ranging from 20- to 1,000-fold. *TB Profiler* can also be downloaded and run locally in a Unix environment [37].

## Discussion

The emergence and amplification of resistance to anti-tuberculosis drugs has created a need for improved detection tools to guide treatment options for patients with MDR-TB, XDR-TB and post XDR (TDR-TB) disease. Molecular-based drug-susceptibility tests are more rapid and microbiologically safe compared to phenotypic assays. Nonetheless, rapid molecular assays are currently limited. *GeneXpert* (Cepheid) tests only for rifampicin resistance, the sensitivity of *GenoType MTBDRplus* (Hain Life-Science) for the detection of isoniazid resistance is reported to be approximately 80% to 90% [38,39] and the *GenoType MTBDRsl* assay performs inadequately for fluoroquinolones, aminoglycosides and ethambutol (reported sensitivities of 87% to 89%, 21% to 100% and 39% to 57%, respectively) [40,41]. Whole-genome sequencing has the potential to determine the full antibiogram if the genetic determinants of antibiotic resistance are known [15-17,42]. However, *M. tuberculosis* sequencing has mainly been performed from cultures and sequencing directly from clinical specimens such as sputum still needs to be optimised. Compared to Sanger sequencing that requires multiple sequencing reactions to cover the various drug resistance loci, whole-genome sequencing has the ability to characterise all nucleotide positions in a single experiment. The depth of next generation sequencing, where each loci is examined numerous times (typically 100-fold coverage) provides capacity to detect genetically mixed bacterial populations (hetero-resistance) [43].

We have compiled and released a mutation library for *M. tuberculosis* drug resistance [22]. By comparing *in silico* drug resistance predictions to conventional phenotypic results, we have demonstrated that our library is more accurate than current commercial molecular tests and alternative mutation databases. Combining the mutation library with a rapid detection tool for whole sequencing data [34], we have demonstrated the potential for using next generation sequencing for detecting drug resistance.

*In silico* validation of the mutation library demonstrated high sensitivity for detecting resistance to RMP, with the majority of resistance mutations found in a single region of the *rpo*B gene [44]. Unsurprisingly, the mutation analysis was less reliable for drugs with more complex modes of action and where knowledge of the genetic basis of resistance is less complete (for example, PZA, ETH and EMB). Still, our curated library was more accurate during *in silico* analysis for MDR and XDR-TB than the commercial line probe assays, in addition to assessing a greater number of drugs. Improved sensitivity was reported for INH, AMK, EMB, PZA and KAN (Figure 2 and Additional file 1: Figure S3). The inferred diagnostic performance from whole genome sequences for the commercial tests may be overestimated, as in a real scenario these tests have low detection limits and are unable to differentiate synonymous from non-synonymous amino acid changes [11].

A limiting factor for this study is the reliability of culture-based susceptibility testing methods, particularly those for EMB and PZA, and the lack of a consensus reference standard with which to compare new tests. Future studies should be encouraged to adopt standardised quantitative phenotypic assays [45]. DST is particularly problematic for PZA [46] and false resistance results are not uncommon [46]. The *pnc*A gene (involved in resistance to PZA) is one of the most polymorphic genes in the *M. tuberculosis* genome and attempts to increase sensitivity by including additional SNPs resulted in a reduction in specificity. Further work is needed to determine additional resistance polymorphisms, including validation of putative markers with high quality phenotypic and clinical data. It should be noted that high positive predictive values are crucial for drug resistance tests where the consequence of a false positive may be unnecessary treatment with drugs of high toxicity and prolonged isolation in dedicated containment facilities. Although an important increase in sensitivity was achieved for EMB (88.7%), the specificity of 81.7% is poor. These results concur with suggestions that degrees of resistance to EMB may be acquired through mutations in multiple loci, some of which are currently unknown [47]. Although current knowledge does not allow EMB resistance to be predicted with high precision, known mutations may be used to identify strains predisposed to developing high-level resistance. Our results demonstrate the considerable cross-resistance between the fluoroquinolones. Minimal inhibitory concentrations (MIC) can vary for these drugs and information on specific polymorphisms may influence dosing levels [48].

The poor specificity obtained for CAP and EMB may be explained in terms of the high MIC used to classify strains as clinically resistant or susceptible. Strains having MIC values slightly below this cutoff have genetically detectable resistance mechanisms but will falsely be identified as susceptible [45,49]. Low specificity was also obtained for MOX (68.7%) as opposed to that of OFX (94.9%) using the same fluoroquinolones resistance markers (that is, *gyrA* and *gyrB* mutations). Mutations in *gyrA* and *gyrB* confer resistance to fluoroquinolones, albeit not at the same level, with MOX normally presenting the lowest MIC values in the group followed by levofloxacin, in contract with the higher levels of resistance observed for OFX and ciprofloxacin [50]. Strains having the same fluoroquinolones resistance-conferring mutations are therefore more likely to be regarded as sensitive phenotypically (false positives) for MOX leading to lower specificity values. However, caution should be exercised when considering the MOX data as few phenotypic results were available and the uncertainty of analysis is reflected in the wide confidence intervals reported.

The accuracy of the mutation analysis was observed to vary by geographic region (Additional file 1: Figure S4). Geographic disparities in the frequency of drug resistant SNPs may reflect local treatment strategies and the clonal nature of tuberculosis transmission and therefore be the result of local microevolution. It has previously been suggested that emergence of resistance in *M. tuberculosis* is associated with bacterial lineage. However, such conclusions cannot be drawn from the present study, as sampling strategies were not appropriate to such analysis.

Not all drugs used in the treatment of tuberculosis were included in this study. Drugs were omitted either because insufficient susceptibility data were available (that is, PAS, LZD, CFZ and BDQ) or because the mechanism of action remains obscure and SNPs to predict resistance have yet to be systematically identified (for example, cycloserine). A major advantage of the whole genome approach is that all data are captured and additional loci can easily be incorporated in the mutation library. Future work should assess the diagnostic accuracy of drug resistance mutations identified for PAS, LZD, CFZ and BDQ in clinical specimens.

Previous studies on discrepancies between mutation and culture-derived resistant phenotypes suggest that molecular assessment may eventually become the reference standard for some drugs [51,52]. We have demonstrated rapid analysis of whole genome sequence data to provide the genotype and predict resistance to 11 anti-TB drugs. In the absence of whole genome sequencing technology, which is still prohibitive in low-resource settings, drug resistance markers can be detected using alternative genotyping platforms, such as multiplex ligation-dependent probe amplification (MLPA) assays [53] or multiplexed oligonucleotides ligation PCR [54]. The presented curated database will facilitate the development of more accurate molecular drug-susceptibility tests.

Rapid determination of strain-specific and drug resistance mutations will be beneficial for therapeutic selection, clinical management of patients and implementation of infection control measures. The free-to-use *TB Profiler* prototype is available for a research setting, and further studies are needed to assess its performance for clinical use.

## Conclusion

We have constructed an on-line software tool and methodology that provides rapid analysis of genome sequence data to describe the lineage of the *M. tuberculosis* strain under test and predict resistance to 11 anti-TB drugs. The tool refers to a library comprising 1,325 mutations that is the most comprehensive and accurate such data source yet reported. In addition to providing information about a greater number of drugs, a whole genome approach has the potential to improve detection sensitivity for drugs such as isoniazid over the currently available molecular tests. The ability to analyse raw sequence data and extract information of clinical relevance in a few minutes would render whole genome analysis faster than current phenotypic testing methods. Accelerated access to tailored treatment could improve cure rates and reduce exposure to ineffective toxic drugs, improving the patient experience and facilitating compliance. The analytical methodology described is flexible to allow moderation of the library to encompass novel mutations and incorporate new drugs should the need arise.

## Additional file

Additional file 1: Table S1. Sources of *M. tuberculosis* drug resistance polymorphisms used to curate a mutation database. **Table S2.** Phylogenetic SNPs not included in the curated database. **Table S3.** Summary of *Mycobacterium tuberculosis* whole genome sequence datasets used in this study. **Table S4.** Novel mutations identified by *TB profiler.* **Table S5.** Diagnostic performance of TB profiler compared to KvarQ method. **Figure S1.** Circos plots summarising drug resistant associated genes and mutations in the curated library for anti-tuberculosis drugs. **Figure S2.** The *TB profiler* tool (http://tbdr.lshtm.ac.uk) - Screenshot of *TB profiler* input page. **Figure S3.** Diagnostic performance of the curated library compared to alternative drug resistance mutation databases, using phenotype drug susceptibility data as the reference standard. **Figure S4.** Diagnostic accuracy across populations.

## Abbreviations

AMK: amikacin
BDQ: bedaquiline
CAP: capreomycin
CFZ: clofazimine
DR: Drug resistance
DST: Drug Susceptibility testing
EMB: ethambutol
ETH: ethionamide
INH: isoniazid
KAN: kanamycin
LPA: Line Probe Assays
LZD: linezolid
MDR-TB: Multi drug-resistant Tuberculosis
MIC: Minimal inhibitory concentrations
MLPA: ligation-dependent probe amplification
MOX: moxifloxacin
indels: insertion and deletions
OFX: ofloxacin
PAS: para-aminosalicylic acid
PZA: pyrazinamide
RMP: rifampicin
SNP: single nucleotide polymorphism
STR: streptomycin
TB: tuberculosis
TDR-TB: totally drug resistant tuberculosis, XDR-TB, extensively drug resistant tuberculosis
WHO: World Health Organization
