# Supplementary material for: Rapid determination of anti-tuberculosis drug resistance from whole-genome sequences
Source: Genome Med. 2015 May 27;7(1):51. doi: 10.1186/s13073-015-0164-0 (PMC4446134; doi:10.1186/s13073-015-0164-0)
Supplement: Additional file 1: Table S1. — Sources of M. tuberculosis drug resistance polymorphisms used to curate a mutation database. Table S2. Phylogenetic SNPs not included in the curated database. Table S3. Summary of Mycobacterium tuberculosis whole genome sequence datasets used in this study. Table S4. Novel mutations identified by TB profiler. Table S5. Diagnostic performance of TB profiler compared to KvarQ method. Figure S1. Circos plots summarising drug resistant associated genes and mutations in the curated library for anti-tuberculosis drugs. Figure S2. The TB profiler tool (http://tbdr.lshtm.ac.uk) - Screenshot of TB profiler input page. Figure S3. Diagnostic performance of the curated library compared to alternative drug resistance mutation databases, using phenotype drug susceptibility data as the reference standard. Figure S4. Diagnostic accuracy across populations. [file 13073_2015_164_MOESM1_ESM.pdf]

SUPPLEMENTARY MATERIAL

Supplementary Table 1. Sources of *M. tuberculosis* drug resistance polymorphisms used to curate a mutation database.

Source/reference	No. of mutations	Type of article
TBDreaMDB (Sandgren et al. 2009)	990	Database
MUBII-TB-DB (Flandrois, Lina, and Dumitrescu 2014)	144	Database
Ajbani *et al.* 2012 – MTBDRsl mutations	18	Test
Ando *et al.* 2010	13	Novel mutations
Andries et al. 2014	5	Novel locus
Beckert et al. 2012	1	Novel locus
Boonaiam *et al.* 2010	16	Novel mutations
Brossier *et al.* 2011	17	Novel mutations
DeBarber *et al.* 2000	8	Novel locus
Engström *et al.* 2012	55	Test
Georghiou *et al.* 2012	26	Review
Helb *et al.* 2010 – XpertMTBRIF mutations	21	Test
Hartkoorn, Uplekar, and Cole 2014	2	Novel locus
Hillemann, Rüsch-Gerdes, and Richter 2008	2	Novel locus
Jin *et al.* 2012 – MTBDRplus mutations	9	Test
Jnawali *et al.* 2013	154	Novel mutations
Liu *et al.* 2013	17	Test
Maruri *et al.* 2012	32	Review
Morlock & Metchock 2003	7	Novel mutations
Moure *et al.* 2013	17	Test
Nebenzahl-Guimaraes *et al.* 2013	52	Review
Safi *et al.* 2013	15	Novel locus
Sekiguchi *et al.* 2007	13	Test
Shi *et al.* 2013	9	Test
Shi et al. 2014	7	Novel locus
Slayden & Barry 2000	33	Review
Stoffels *et al.* 2012	82	Novel mutations
Tan *et al.* 2013	38	Novel mutations
Wang *et al.* 2013	13	Test
Zhao et al. 2014	11	Novel mutations
Zhang *et al.* 2013	64	Novel mutations
S. Zhang et al. 2013	5	Novel locus
Zhang et al. 2014	37	Novel mutations
Zimenkov *et al.* 2013	54	Test

In addition to published articles two databases were consulted, *TBDreaMDB* (Sandgren et al. 2009) and *MUBII-TB-DB* (Flandrois et al. 2014). Lineage specific mutations and polymorphisms without sound phenotypic data supporting their association with resistance were discarded (Supplementary Table 2). See SupplementaryData1.xlsx (http://pathogenseq.lshtm.ac.uk/rapiddrdata) for the full list of drug resistance mutations.

Supplementary Table 2. Phylogenetic SNPs not included in the curated database

Gene	Chromosome position	Nucleotide change	Codon change	Codon number *	Amino acid change	Locus Tag	Lineage/clade**	Source***
rpoC	765150	G/A	GGG/GAG	594	G/E	*Rv0668*	4.1	(Coll et al. 2014)
rpoC	763884	C/T	GCC/GTC	172	A/V	*Rv0668*	1	(Coll et al. 2014)
rpoC	763886	C/A	CGG/AGG	173	R/R	*Rv0668*	1	(Coll et al. 2014)
rpoC	765171	C/T	CCG/CTG	601	P/L	*Rv0668*	1.1	(Coll et al. 2014)
rpoC	765230	G/A	GCG/ACG	621	A/T	*Rv0668*	1.1.3	(Coll et al. 2014)
rpoC	764995	C/G	GCC/GCG	542	A/A	*Rv0668*	4.3	(Coll et al. 2014)
rpoC	764013	A/C	GAG/GCG	215	E/A	*Rv0668*	7	(Coll et al. 2014)
rpoC	766955	G/A	GAG/AAG	1196	E/K	*Rv0668*	7	(Coll et al. 2014)
rpoA	3877553	C/T	GAG/AAG	319	E/K	*Rv3457c*	4.1.1.1 sub-clade	(Coll et al. 2014)
rpoB	763031	T/C	GCT/GCC	1075	A/A	*Rv0667*	4	(Coll et al. 2014)
rpoB	760115	C/T	GAC/GAT	103	D/D	*Rv0667*	4.1.2 sub-clade	(Coll et al. 2014)
rpoB	762434	T/G	GGT/GGG	876	G/G	*Rv0667*	3	(Coll et al. 2014)
pncA	2289047	G/A	TCC/TCT	65	S/S	*Rv2043c*	3	(Coll et al. 2014);(Feuerriegel et al. 2014)
pncA	2289104	T/C	GCA/GCG	46	A/A	*Rv2043c*	*M. canettii*	(Feuerriegel et al. 2014)
gid	4408156	A/C	CTT/CGT	16	L/R	*Rv3919c*	4.3	(Coll et al. 2014)
gid	4407588	T/C	GCA/GCG	205	A/A	*Rv3919c*	4	(Coll et al. 2014)
gid	4407780	C/T	GCG/GCA	141	A/A	*Rv3919c*	1.1.3	(Coll et al. 2014)
gid	4407873	C/A	GTG/GTT	110	V/V	*Rv3919c*	1	(Coll et al. 2014)
gid	4407927	T/G	GAA/GAC	92	E/D	*Rv3919c*	2.2	(Coll et al. 2014)
embB	4249408	G/A	CCG/CCA	965	P/P	*Rv3795*	4 sub-clade	(Coll et al. 2014)
embB	4247646	A/G	GAG/GGG	378	E/G	*Rv3795*	Ancient lineages	(Coll et al. 2014);(Feuerriegel et al. 2014)
embB	4246864	C/T	GTC/GTT	117	V/V	*Rv3795*	BOV_AFRI	(Coll et al. 2014)
embB	4246930	G/C	CAG/CAC	139	Q/H	*Rv3795*	4.1.1.2	(Coll et al. 2014)
embB	4248115	C/T	GAC/GAT	534	D/D	*Rv3795*	2.2.1.1	(Coll et al. 2014)
embB	4249012	G/A	CTG/CTA	833	L/L	*Rv3795*	4.4.1.2	(Coll et al. 2014)
embB	4249732	C/G	GCC/GCG	1073	A/A	*Rv3795*	4.7	(Coll et al. 2014)
embB	4248073	C/T	ACC/ACT	520	T/T	*Rv3795*	7	(Coll et al. 2014)
embB	4247590	A/G	CTA/CTG	359	L/L	*Rv3795*	*M. canettii*	(Feuerriegel et al. 2014)
embB	4247815	C/T	GCC/GCT	434	A/A	*Rv3795*	*M. canettii*	(Feuerriegel et al. 2014)
embB	4248195	A/G	AAG/AGG	561	K/R	*Rv3795*	*M. canettii*	(Feuerriegel et al. 2014)
embB	4248206	A/G	AGC/GGC	565	S/G	*Rv3795*	*M. canettii*	(Feuerriegel et al. 2014)
embB	4248439	C/T	ACC/ACG	642	T/T	*Rv3795*	*M. canettii*	(Feuerriegel et al. 2014)
embA	4243346	A/G	CAA/CAG	38	Q/Q	*Rv3794*	4 sub-clade	(Coll et al. 2014);(Feuerriegel et al. 2014)
embA	4245969	C/T	CCG/TCG	913	P/S	*Rv3794*	1	(Coll et al. 2014)
embA	4243848	G/A	GTG/ATG	206	V/M	*Rv3794*	1.1	(Coll et al. 2014)
embA	4244220	C/T	CTG/TTG	330	L/L	*Rv3794*	1.2.1	(Coll et al. 2014)
embA	4243460	C/T	TGC/TGT	76	C/C	*Rv3794*	2.2	(Coll et al. 2014);(Feuerriegel et al. 2014)
embA	4246088	A/G	CAA/CAG	952	Q/Q	*Rv3794*	2.1	(Coll et al. 2014)
embA	4246508	G/A	GCG/GCA	1092	A/A	*Rv3794*	4.4.2	(Coll et al. 2014)
embA	4245055	C/A	ACC/AAC	608	T/N	*Rv3794*	4.6.1.2	(Coll et al. 2014)
embA	4243377	A/C	AGC/CGC	49	S/R	*Rv3794*	*M. canettii*	(Feuerriegel et al. 2014)
embA	4243690	T/C	ATG/ACG	153	M/T	*Rv3794*	*M. canettii*	(Feuerriegel et al. 2014)
embC	4240897	C/G	CGC/CGG	345	R/R	*Rv3793*	4.1.1.1	(Coll et al. 2014)
embC	4242803	G/C	GTG/CTG	981	V/L	*Rv3793*	4.1	(Coll et al. 2014);(Feuerriegel et al. 2014)
embC	4241042	A/G	AAC/GAC	394	N/D	*Rv3793*	1	(Coll et al. 2014)
embC	4242075	G/A	CGG/CAG	738	R/Q	*Rv3793*	3	(Coll et al. 2014);(Feuerriegel et al. 2014)
embC	4241562	G/A	CGC/CAC	567	R/H	*Rv3793*	3.1.1	(Coll et al. 2014)
embC	4240172	G/A	GTG/ATG	104	V/M	*Rv3793*	3 sub-clade	(Coll et al. 2014)
embC	4240671	C/T	ACC/ATC	270	T/I	*Rv3793*	Ancient lineages	(Coll et al. 2014);(Feuerriegel et al. 2014)
embC	4242643	C/T	CGC/CGT	927	R/R	*Rv3793*	4.9	(Coll et al. 2014)
embC	4241539	T/C	GGT/GGC	559	G/G	*Rv3793*	4.3 sub-clade	(Coll et al. 2014)

embC	4242883	C/T	CCC/CCT	1007	P/P	*Rv3793*	4.6.2.1	(Coll et al. 2014)
embC	4240153	G/A	TCG/TCA	97	S/S	*Rv3793*	7	(Coll et al. 2014)
embC	4242970	C/T	ACC/ACT	1036	T/T	*Rv3793*	*M. bovis - M. caprae*	(Feuerriegel et al. 2014)
gyrA	7892	G/A	CTG/CTA	197	L/L	*Rv0006*	4.5	(Coll et al. 2014)
gyrA	7539	A/G	ACC/GCC	80	T/A	*Rv0006*	4.6.1	(Coll et al. 2014);(Feuerriegel et al. 2014)
gyrA	9304	G/A	GGC/GAC	668	G/D	*Rv0006*	Clade of 4.7, 4.8 and 4.9	(Coll et al. 2014)
gyrA	7585	G/C	AGC/ACC	95	S/T	*Rv0006*	Clade of 4.7, 4.8 and 4.9	(Coll et al. 2014);(Feuerriegel et al. 2014)
gyrA	8135	C/T	GTC/GTT	278	V/V	*Rv0006*	4.8 sub-clade	(Coll et al. 2014)
gyrA	8978	C/T	TTC/TTT	559	F/F	*Rv0006*	4.9 sub-clade	(Coll et al. 2014)
gyrA	8452	C/T	GCA/GTA	384	A/V	*Rv0006*	1	(Coll et al. 2014)
gyrA	8188	T/C	CTG/CCG	296	L/P	*Rv0006*	1.1.3 sub-clade	(Coll et al. 2014)
gyrA	8040	G/A	GGC/AGC	247	G/S	*Rv0006*	4.3.3	(Coll et al. 2014)
gyrA	9143	T/C	ATT/ATC	614	I/I	*Rv0006*	Ancient lineages	(Coll et al. 2014)
gyrA	9260	G/C	CTG/CTC	653	L/L	*Rv0006*	1.2.1	(Coll et al. 2014)
gyrA	9611	C/T	GAC/GAT	770	D/D	*Rv0006*	3.1.2.2	(Coll et al. 2014)
gyrA	9566	C/T	TAC/TAT	755	Y/Y	*Rv0006*	5	(Coll et al. 2014)
gyrA	8876	C/T	TAC/TAT	525	Y/Y	*Rv0006*	7	(Coll et al. 2014)
gyrB	6140	G/T	GTG/TTG	301	V/L	*Rv0005*	4.3.4.2 sub-clade	(Coll et al. 2014)
gyrB	6124	C/T	GCC/GCT	295	A/A	*Rv0005*	1.1.2	(Coll et al. 2014)
gyrB	6112	G/C	ATG/ATC	291	M/I	*Rv0005*	1	(Coll et al. 2014)
gyrB	6446	G/T	GCG/TCG	403	A/S	*Rv0005*	Clade of *M. bovis*, 5 and 6	(Coll et al. 2014)
gyrB	6817	G/A	AAG/AAA	526	K/K	*Rv0005*	4.3.4 sub-clade	(Coll et al. 2014)
katG	2154724	C/A	CGG/CTG	463	R/L	*Rv1908c*	4	(Coll et al. 2014);(Feuerriegel et al. 2014)
katG	2155503	G/A	ACC/ACT	203	T/T	*Rv1908c*	BOV_AFRI	(Coll et al. 2014)
fabG1 promoter	1673338	G/A	-	-102	-	*Rv1482c-Rv1483*	5 sub-clade	(Coll et al. 2014);(Feuerriegel et al. 2014)
inhA	1674434	T/C	GTG/GCG	78	V/A	*Rv1484*	6	(Coll et al. 2014);(Feuerriegel et al. 2014)
inhA	1674883	A/G	ATC/GTC	228	I/V	*Rv1484*	1.1.3 sub-clade	(Coll et al. 2014)
inhA	1674816	T/G	GGT/GGC	205	G/G	*Rv1484*	*M. canettii*	(Feuerriegel et al. 2014)
inhA	1674520	C/T	CCG/TCG	107	P/S	*Rv1484*	*M. pinnipedii*	(Feuerriegel et al. 2014)
ahpC	2726210	T/C	ATT/ATC	6	I/I	*Rv2428*	4.1.1.3 sub-clade	(Coll et al. 2014)
ahpC promoter	2726105	G/A	-	-88	-	*Rv2427A-Rv2428*	3	(Coll et al. 2014);(Feuerriegel et al. 2014)
kasA	2518919	G/A	GGT/AGT	269	G/S	*Rv2245*	4.3.3	(Coll et al. 2014)
embR	1417019	C/T	TGC/TAC	110	C/Y	*Rv1267c*	1	(Coll et al. 2014)
embR	1416410	A/C	CTG/CGG	313	L/R	*Rv1267c*	4.6.1.2	(Coll et al. 2014)
embR	1416702	A/G	TAC/CAC	216	Y/H	*Rv1267c*	4.6.2.1	(Coll et al. 2014)
embR	1416977	T/C	CAC/CGX	124	H/R	*Rv1267c*	7	(Coll et al. 2014)
rpsA	1834177	A/C	CGA/CGC	212	R/R	*Rv1630*	2	(Coll et al. 2014);(Feuerriegel et al. 2014)
rpsA	1834836	T/C	ATG/ACG	432	M/T	*Rv1630*	4.3.3 sub-clade	(Coll et al. 2014)
rpsA	1834859	G/A	GCG/ACG	440	A/T	*Rv1630*	*M. bovis* sub-clade	(Coll et al. 2014);(Feuerriegel et al. 2014)
rpsA	1834916	A/C	ACC/CCC	459	T/P	*Rv1630*	7	(Coll et al. 2014)
rpsA	1833554	A/G	ACC/GCC	5	T/A	*Rv1630*	*M. canettii*	(Feuerriegel et al. 2014)
rpsA	1833568	G/C	CCG/CCC	9	P/P	*Rv1630*	*M. canettii*	(Feuerriegel et al. 2014)
rpsA	1834169	A/G	ACC/GCC	210	T/A	*Rv1630*	*M. canettii*	(Feuerriegel et al. 2014)
rpsA	1834912	A/G	GAA/GAG	457	E/E	*Rv1630*	*M. canettii*	(Feuerriegel et al. 2014)
ethA	4326676	G/C	AGC/AGG	266	S/R	*Rv3854c*	2.1.1. sub-clade	(Coll et al. 2014)
ethA	4327103	C/T	GGC/GAC	124	G/D	*Rv3854c*	5 sub-clade	(Coll et al. 2014)
ethA	4326928	G/A	GGC/GGT	182	G/G	*Rv3854c*	5 sub-clade	(Coll et al. 2014)
ethA	4326439	G/T	AAC/AAA	345	N/K	*Rv3854c*	1.2.2	(Coll et al. 2014)
ethA	4326148	C/A	TCG/TCT	442	S/S	*Rv3854c*	1.2.2 sub-clade	(Coll et al. 2014)
ethA	4327450	G/A	GTC/GTT	8	V/V	*Rv3854c*	1.2.2 sub-clade	(Coll et al. 2014)
ethA	4326176	T/G	GAG/GCG	433	E/A	*Rv3854c*	3.1.2.2	(Coll et al. 2014)
ethA	4326739	G/C	CGC/CGG	245	R/R	*Rv3854c*	4.6.2.2	(Coll et al. 2014)
ethA	4328004	G/A	GTG/GTA	152	V/V	*Rv3854c*	4.6.2.2	(Coll et al. 2014)

ethR	4328127	G/C	TCG/TCC	193	S/S	*Rv3855*	4.3.4.2 sub-clade	(Coll et al. 2014)
rrs	1472337	C/T	-	492	-	*rrs*	4.3.2	(Coll et al. 2014)
rrl	1474001	C/T	-	344	-	*rrl*	4.8	(Coll et al. 2014)
tlyA	1918281	A/C	GGA/GGC	114	G/G	*Rv1694*	7	(Coll et al. 2014)
folC	2746340	G/A	GCG/GTG	420	A/V	*Rv2447c*	4.3.3 sub-clade	(Coll et al. 2014)
thyA	3073868	T/C	ACC/GCC	202	T/A	*Rv2764c*	4.3	(Coll et al. 2014);(Feuerriegel et al. 2014)
rplC	801166	G/A	GGC/AGC	129	G/S	*Rv0701*	1.1.3 sub-clade	(Coll et al. 2014)

* The codon number is specified for protein coding regions. The gene coordinate rather than the codon number is used for RNA coding genes (i.e. *rrs* and *rrl*) and promoters. ** Lineage/clade refers to the branch in the MTBC phylogeny where the phylogenetic SNP was originated. Refer to Coll et al. 2014 for lineage and sub-lineage nomenclature. *** Two sources were used to extract phylogenetic SNPs: (Coll et al. 2014) and (Feuerriegel et al. 2014)

Supplementary Table 3 Summary of *Mycobacterium tuberculosis* whole genome sequence datasets used in this study

Population (reference)	No. samples	DST method	ENA accession number	Read length	Median depth of coverage	% Pan susceptible	% Any resistance	% MDR-TB	% XDR-TB
China (H. Zhang et al. 2013)	161	Solid medium	SRA065095	100	113	27·3	72·7	72·7	14·3
Karachi, Pakistan (Ali et al. 2015)	42	Agar proportion method	Not available	100	448	11·9	88·1	88·1	0
Karonga, Malawi (Guerra-Assunção et al. 2015)	337	Ratio method	ERP000436	75	183	90·8	9·2	0·6	0
Lisbon & Porto, Portugal (Perdigão et al. 2014)	212	BACTEC 960 MGIT or BACTEC 460	ERP002611*	100	157	21·7	78·3	44·8	12·7
Samara, Russia (Casali and Nikolayevskyy 2012)	21	BACTEC 960 MGIT	ERP000192	50	38	33·3	66·7	52·4	19·0
Vancouver, Canada (Gardy et al. 2011)	19	BACTEC 960 MGIT	SRP002589	50	58	100	0	0	0
**Overall**	**792**				**169**	**53·9**	**46·1**	**33·1**	**6·8**

Supplementary Table 4 Novel mutations identified by *TB profiler*

Drug	DR Candidate Gene	Mutations[a]	Number of samples (Population)	Increased sensitivity[b]
INH	*katG*	G299S, P232S, F408L, D142G, G120S, CTCGGGT2155245C, D189A, D419G	7 (China)	2·3%
	ahpC promoter	C2726136T		
RMP	*rpoB*	S450Stop, CAGCCAGCTG761087C	2 (Pakistan and Portugal)	0·7%
EMB	*embA* promoter	C4243225T, G4243190C, C4243218CTACCATCGAG	7 (6 from China, 1 from Portugal)	4·7%
	embA	G554D, G200S		
	embB	A679T, Y319D, S538P, S412P, N399T		
PZA	*pncA*	V130M, G2289011GT, I133S, G2288786GGCCAAGCCAT (n=2), G2289011GT	7 (6 from Pakistan, 1 from Portugal)	6·4%
	rpsA	Q410R (n=2)		
ETH	*fabG1* promoter	T1673432G, T1673432A	9 samples (4 from China, 5 from Portugal)	5·7%
	inhA	I95L, I194T		
	ethA	CT4326393C, GT4327132G, A4326800AGC, C403R (n=2), Y143Stop, P51S, P149S		

[a]Mutations present in phenotypically resistant samples and absent in susceptible cases, which are not strain-specific or synonymous SNPs.
[b]Increased overall sensitivity to predict phenotypic drug resistance if mutations are added to the curated list.
INH: isoniazid; RMP: rifampicin; EMB: ethambutol; PZA: pyrazinamide; STR: streptomycin; ETH: ethionamide

Supplementary Table 5 Diagnostic performance of TB profiler compared to KvarQ method.

			Curated drug resistance database results			KvarQ results		
Drug	Sample size	Resistant (%)	Sensitivity (95%CI)	Specificity (95%CI)	Accuracy (95%CI)	Sensitivity (95%CI)	Specificity (95%CI)	Accuracy (95%CI)
**INH**	693	305 (44)	92·8 (89·9-95·7)	100 (100-100)	96·8 (95·5-98·1)	86·9 (83·1-90·7)	100 (100-100)	94·2 (92·5-96)
**RMP**	694	264 (38)	96·2 (93·9-98·5)	98·1 (96·8-99·4)	97·4 (96·2-98·5)	95·8 (93·4-98·2)	97·9 (96·5-99·3)	97·1 (95·9-98·4)
**EMB**	484	150 (31)	88·7 (83·6-93·8)	81·7 (77·6-85·8)	83·9 (80·6-87·2)	60·7 (52·8-68·5)	89·2 (85·9-92·5)	80·4 (76·8-83·9)
**STR**	487	225 (46.2)	87·1 (82·7-91·5)	87·1 (86-93·4)	88·5 (85·7-91·3)	80 (74·8-85·2)	96·2 (93·9-98·5)	88·7 (85·9-91·5)
**PZA**	307	110 (35.8)	70·9 (62·4-79·4)	93·9 (90·6-97·2)	85·7 (81·7-89·6)	62·7 (53·7-71·8)	93·9 (90·6-97·2)	82·7 (78·5-87)
**ETH**	334	155 (46.4)	73·6 (66·7-80·5)	93·3 (89·6-97)	84·1 (80·2-88·1)	ND	ND	ND
**MOX**	42	10 (23.8)	60 (29·6-90·4)	68·7 (52·6-84·8)	66·7 (52·4-80·9)	60 (29·6-90·4)	68·75 (52·7-84·8)	66·7 (52·4-80·9)
**OFX**	313	117 (37.4)	85·5 (79·1-91·9)	94·9 (91.8-98)	91·4 (88·3-94·5)	82 (75·1-89)	93·9 (90·5-97·2)	89·5 (86·1-92·8)
**AMK**	193	76 (39.4)	82·9 (74·4-91·4)	98·3 (96-100)	92·2 (88·4-96)	75 (65·3-84·7)	98·3 (95·9-100)	89·1 (84·7-93·5)
**CAP**	358	89 (24.9)	60·7 (50·6-70·8)	90·7 (87·2-94·2)	83·2 (79·4-87·1)	ND	ND	ND
**KAN**	118	118 (37.3)	87·3 (81·3-93·3)	93·4 (89·9-96·9)	91·1 (88-94·3)	54·2 (45·2-63·2)	98·5 (96·8-100)	82 (77·7-86·2)
**MDR**	693	262 (37.8)	91·2 (87·8-94·6)	98·4 (97·2-99·6)	95·8 (94·3-97·3)	88·5 (84·7-92·4)	92·3 (89·8-94·8)	90·9 (88·8-93)
**XDR**	601	54 (9)	75·9 (64·5-87·3)	98·4 (97·3-99·5)	96·3 (94·8-97·8)	31·5 (19·1-43·7)	99·6 (99·1-100)	93·5 (91·5-95·5)

Supplementary Figure 1

Ciros plots summarising drug resistant associated genes and mutations in the curated library for anti-tuberculosis drugs

a) Isoniazid
b) Rifampicin
c) Ethambutol
d) Pyrazinamide
e) Streptomycin
f) Ethionamide
g) Fluoroquinolones
h) Amikacin
i) Capreomycin
j) Kanamycin

Colour-coded bars represent genes involved in drug resistance. Above the bars a grey histogram shows the marker density, as derived from the curated library. Thus, grey areas highlight drug-resistance determining regions in candidate genes, which in some cases span the whole gene (e.g. *katG* or *pncA*) or are confined to a certain region (e.g. *rpoB* or *embB*). Vertical black lines indicate the frequency of markers observed in the studied dataset (n=792). Grey areas may lack these lines if markers in the curated list were not found in the studied populations. Internal black lines show co-occurring markers both within and between genes. The thickness of these lines is proportional to the frequency of the markers that occur concurrently.

a) Isoniazid

294 variable sites, 348 SNPs and 25 indels, in 4 genes and 3 promoters

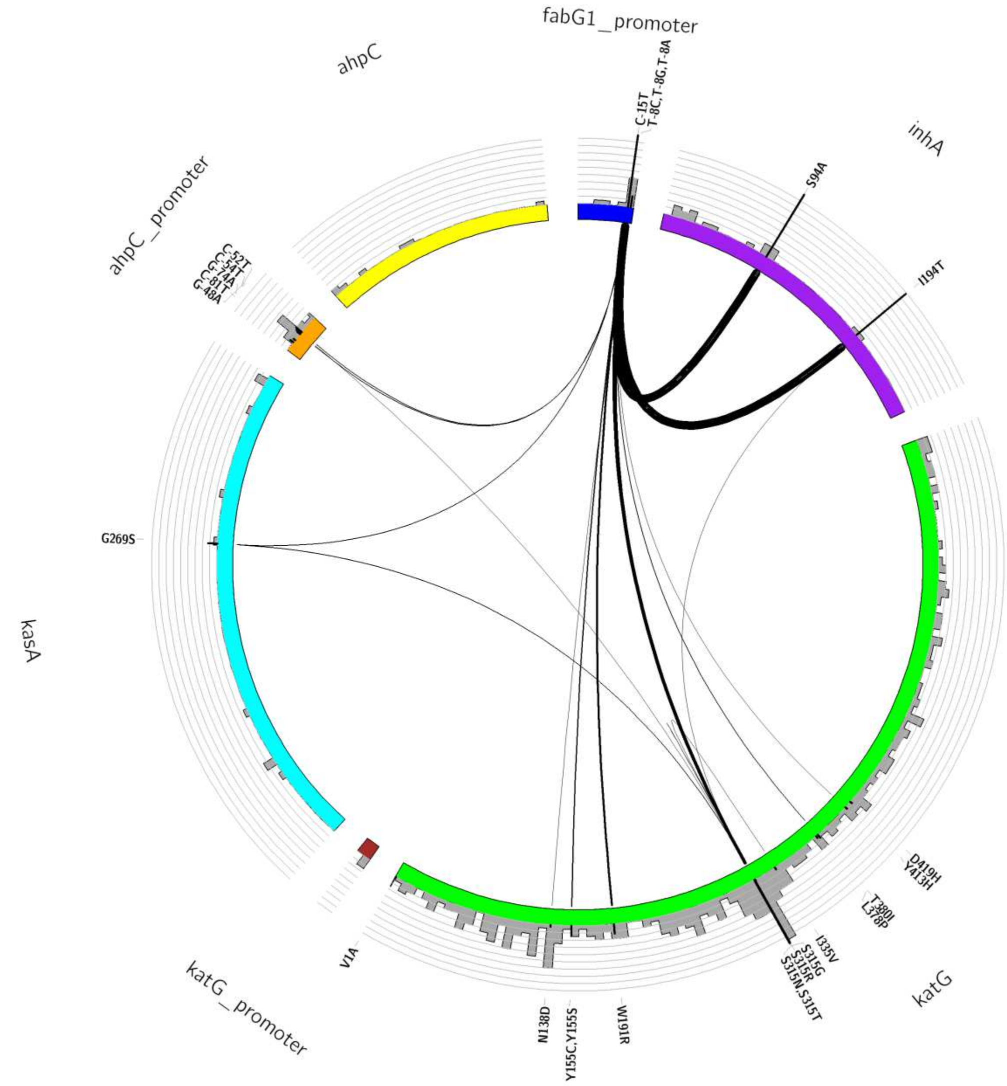

b) Rifampicin

97 variable sites, 143 SNPs and 19 indels, in 2 genes

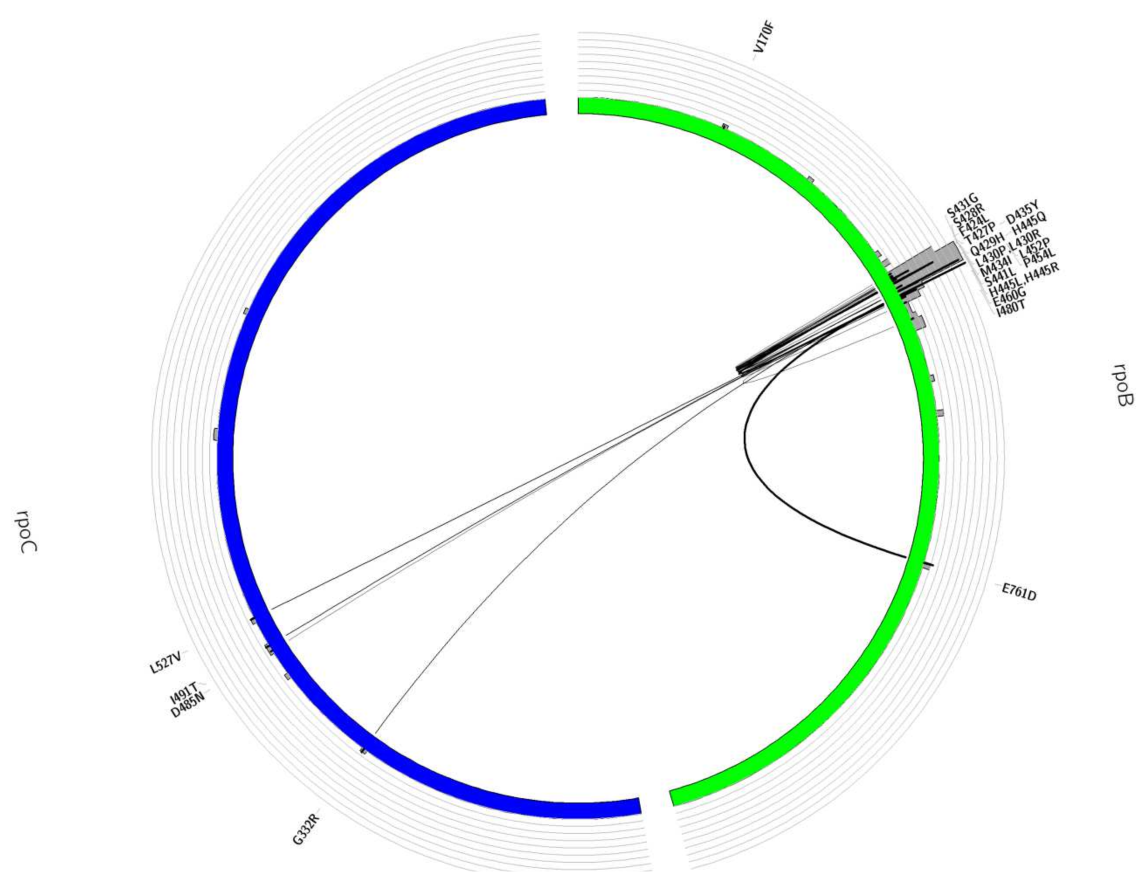

c) Ethambutol

178 variable sites, 211 SNPs and 1 indel, in 4 genes and 1 promoter

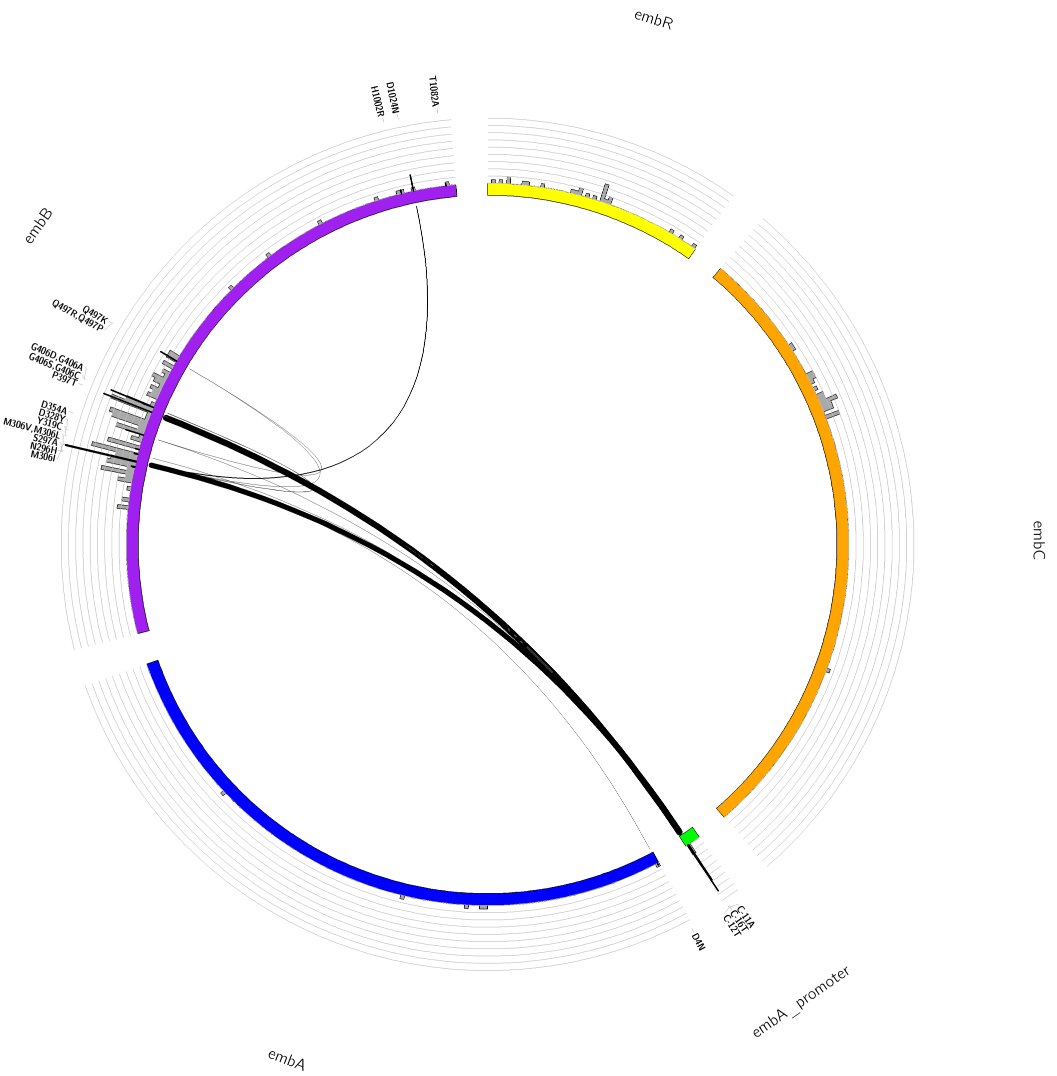

d) Pyrazinamide

222 variable sites, 279 SNPs and 64 indels, in 2 genes and 1 promoter

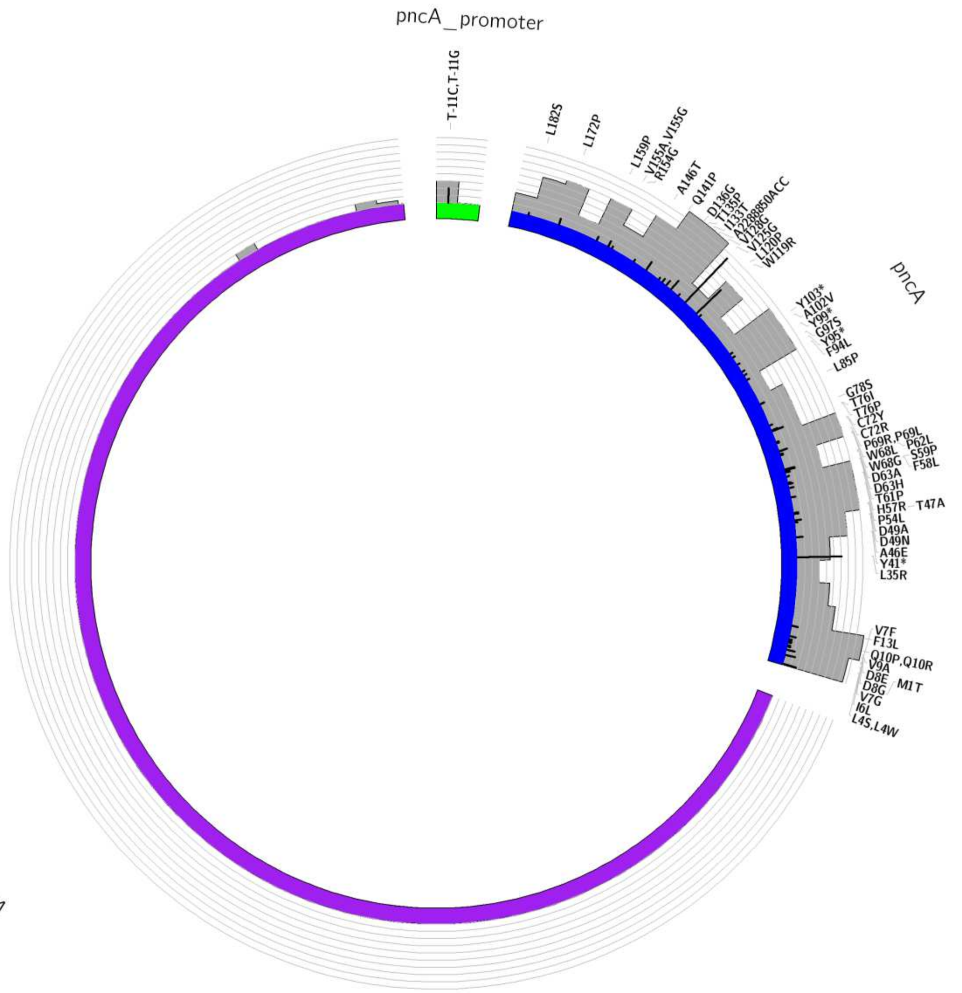

e) Streptomycin

35 variable sites, 44 SNPs, in 2 genes

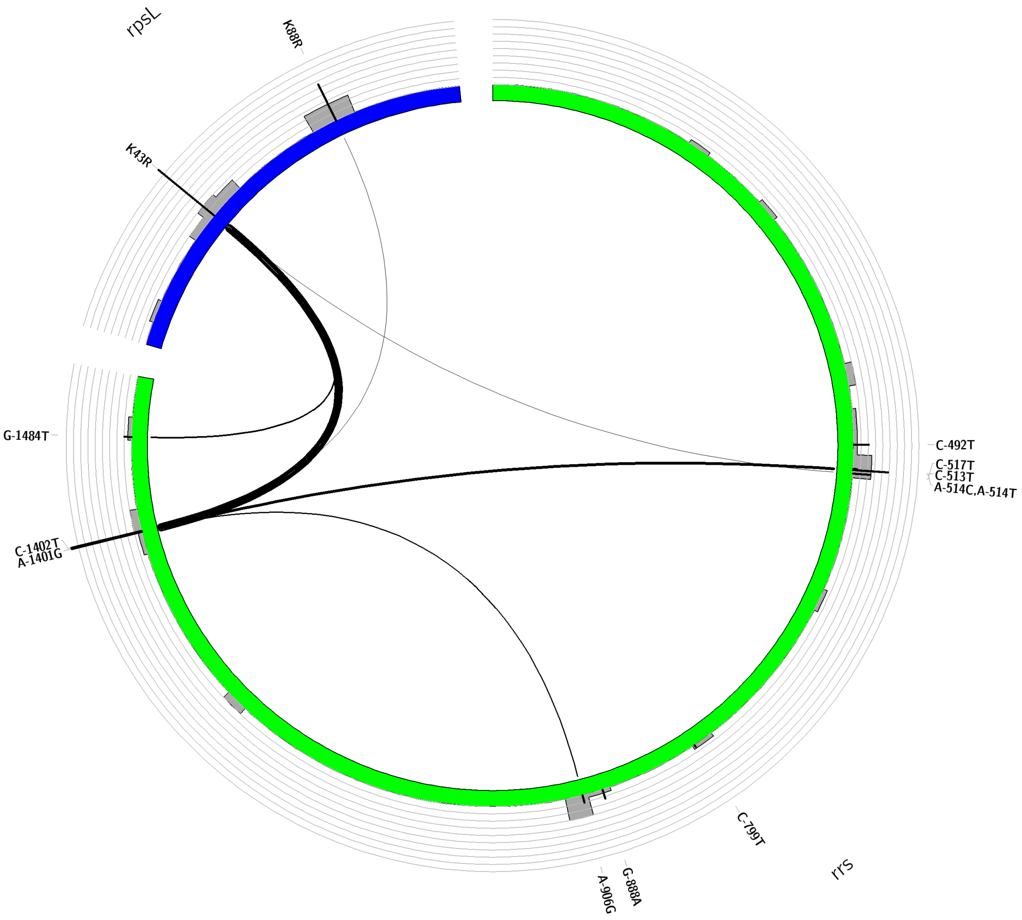

f) Ethionamide

42 variable sites, 39 SNPs and 5 indels, in 3 genes and 1 promoter

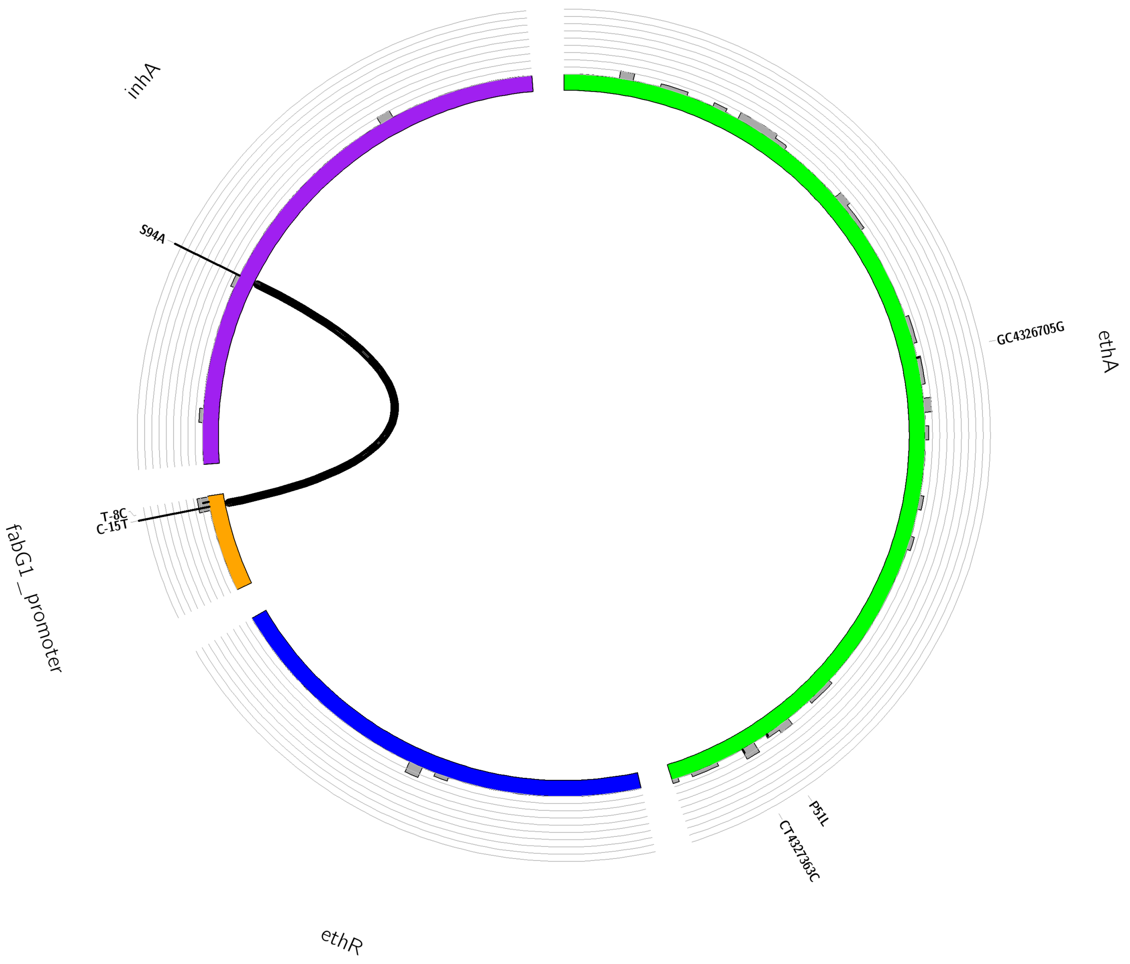

g) Fluoroquinolones (moxifloxacin and ofloxacin)

37 variable sites, 51 SNPs, 2 genes

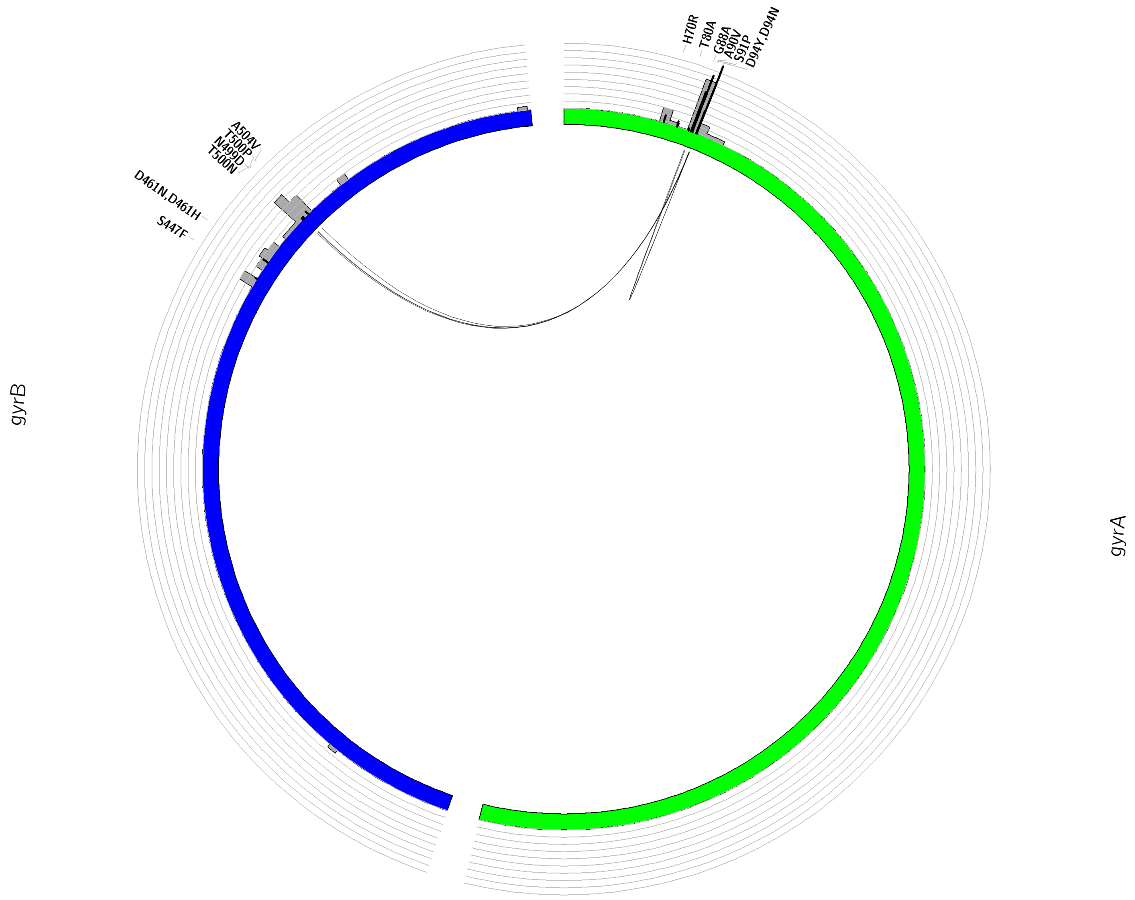

h) Amikacin

8 variable sites, 9 SNPs, in 1 gene

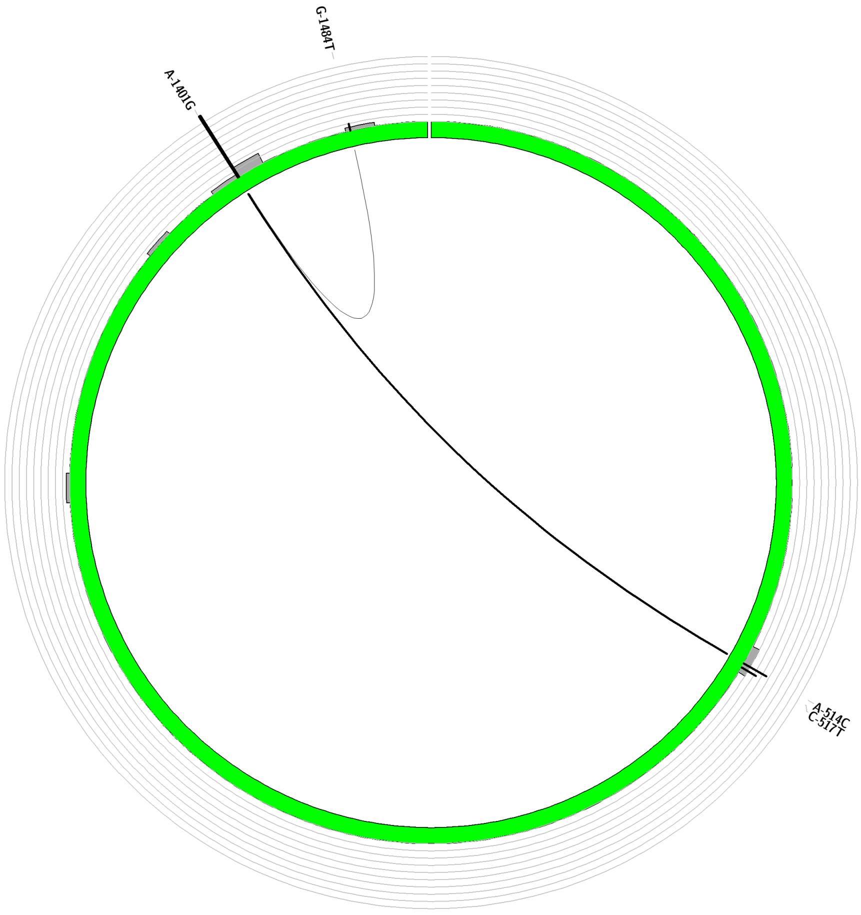

rrs

i) Capreomycin

29 variable sites, 22 SNPs and 10 indels, in 2 genes

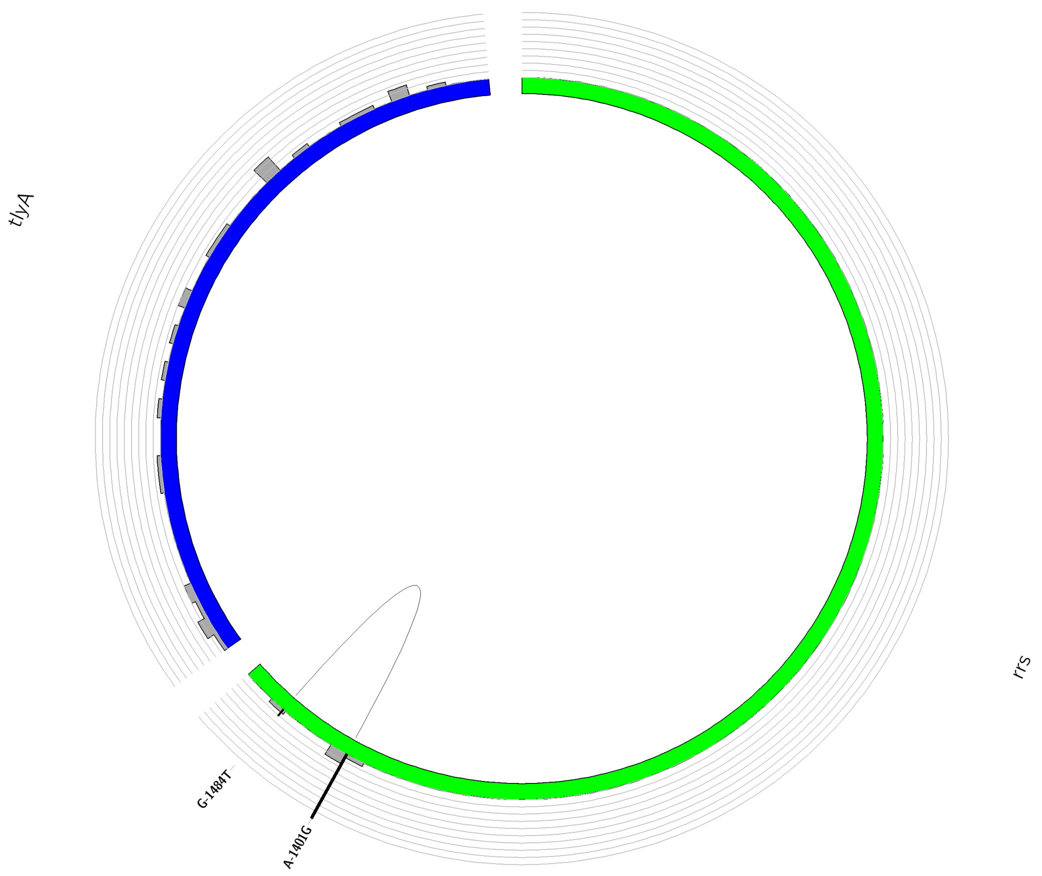

j) Kanamycin

12 variable sites, 14 SNPs, in 1 gene and 1 promoter

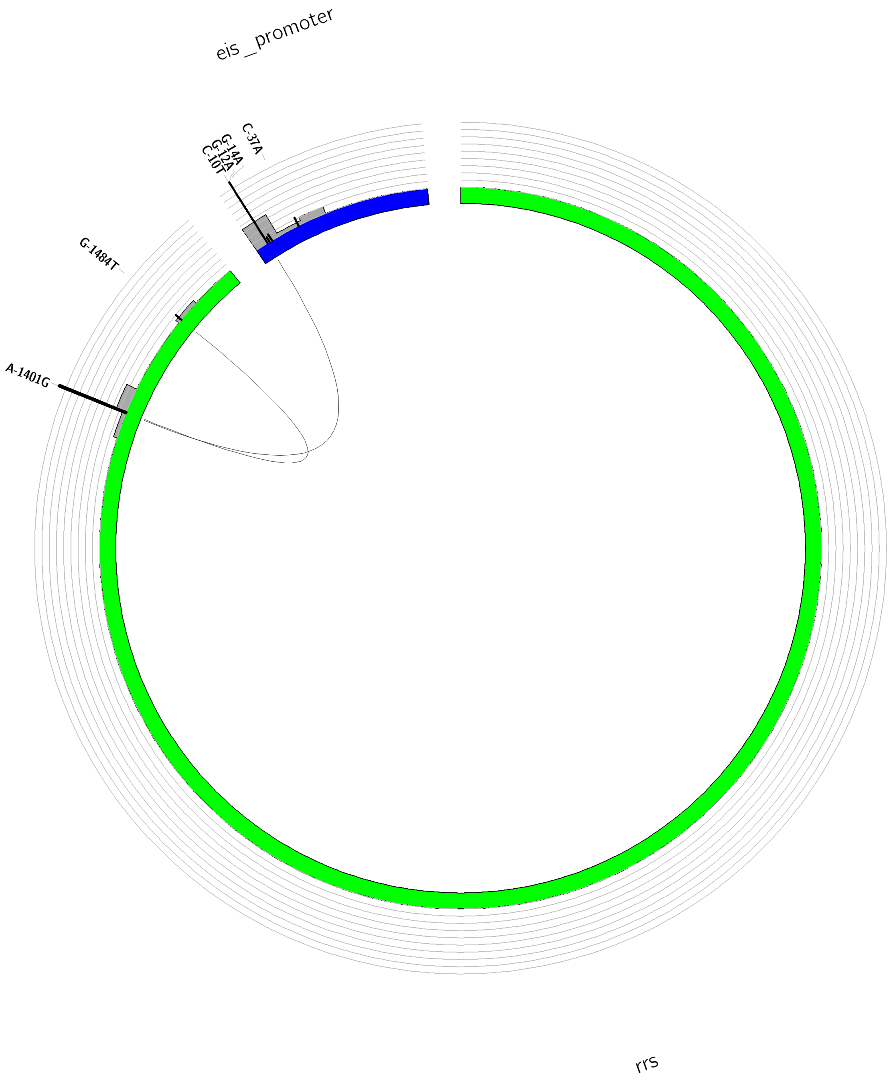

Supplementary Figure 2 The *TB profiler* tool (http://tbdr.lshtm.ac.uk)

Screenshot of *TB profiler* input page

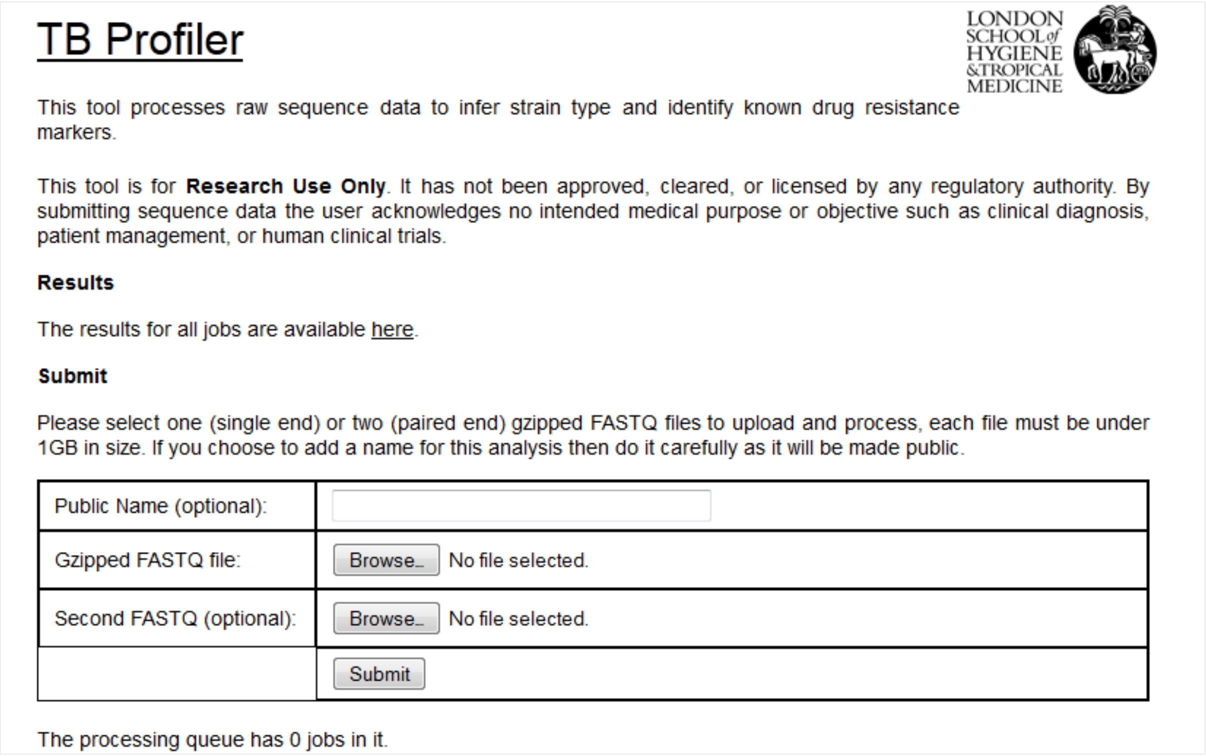

TB Profiler

This tool processes raw sequence data to infer strain type and identify known drug resistance markers.

This tool is for **Research Use Only**. It has not been approved, cleared, or licensed by any regulatory authority. By submitting sequence data the user acknowledges no intended medical purpose or objective such as clinical diagnosis, patient management, or human clinical trials.

Results

The results for all jobs are available here.

Submit

Please select one (single end) or two (paired end) gzipped FASTQ files to upload and process, each file must be under 1GB in size. If you choose to add a name for this analysis then do it carefully as it will be made public.

Public Name (optional):	
Gzipped FASTQ file:	Browse... No file selected.
Second FASTQ (optional):	Browse... No file selected.
	Submit

The processing queue has 0 jobs in it.

Screenshot of *TB profiler* output page: case of an XDR-TB Beijing sample

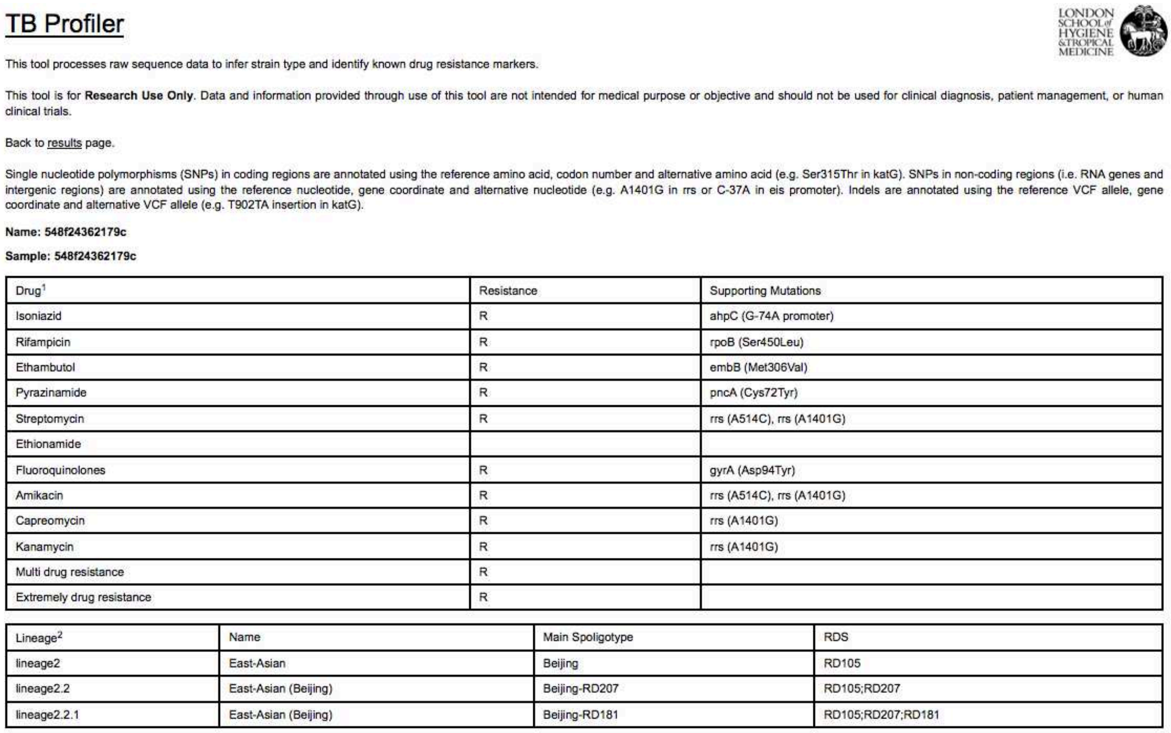

TB Profiler

This tool processes raw sequence data to infer strain type and identify known drug resistance markers.

This tool is for **Research Use Only**. Data and information provided through use of this tool are not intended for medical purpose or objective and should not be used for clinical diagnosis, patient management, or human clinical trials.

Back to results page.

Single nucleotide polymorphisms (SNPs) in coding regions are annotated using the reference amino acid, codon number and alternative amino acid (e.g. Ser315Thr in katG). SNPs in non-coding regions (i.e. RNA genes and intergenic regions) are annotated using the reference nucleotide, gene coordinate and alternative nucleotide (e.g. A1401G in rrs or C-37A in eis promoter). Indels are annotated using the reference VCF allele, gene coordinate and alternative VCF allele (e.g. T902TA insertion in katG).

Name: 548f24362179c

Sample: 548f24362179c

Drug[1]	Resistance	Supporting Mutations
Isoniazid	R	ahpC (G-74A promoter)
Rifampicin	R	rpoB (Ser450Leu)
Ethambutol	R	embB (Met306Val)
Pyrazinamide	R	pncA (Cys72Tyr)
Streptomycin	R	rrs (A514C), rrs (A1401G)
Ethionamide		
Fluoroquinolones	R	gyrA (Asp94Tyr)
Amikacin	R	rrs (A514C), rrs (A1401G)
Capreomycin	R	rrs (A1401G)
Kanamycin	R	rrs (A1401G)
Multi drug resistance	R	
Extremely drug resistance	R	

Lineage[2]	Name	Main Spoligotype	RDS
lineage2	East-Asian	Beijing	RD105
lineage2.2	East-Asian (Beijing)	Beijing-RD207	RD105;RD207
lineage2.2.1	East-Asian (Beijing)	Beijing-RD181	RD105;RD207;RD181

Supplementary Figure 3 Diagnostic performance of the curated library compared to alternative drug resistance mutation databases, using phenotype drug susceptibility data as the reference standard.

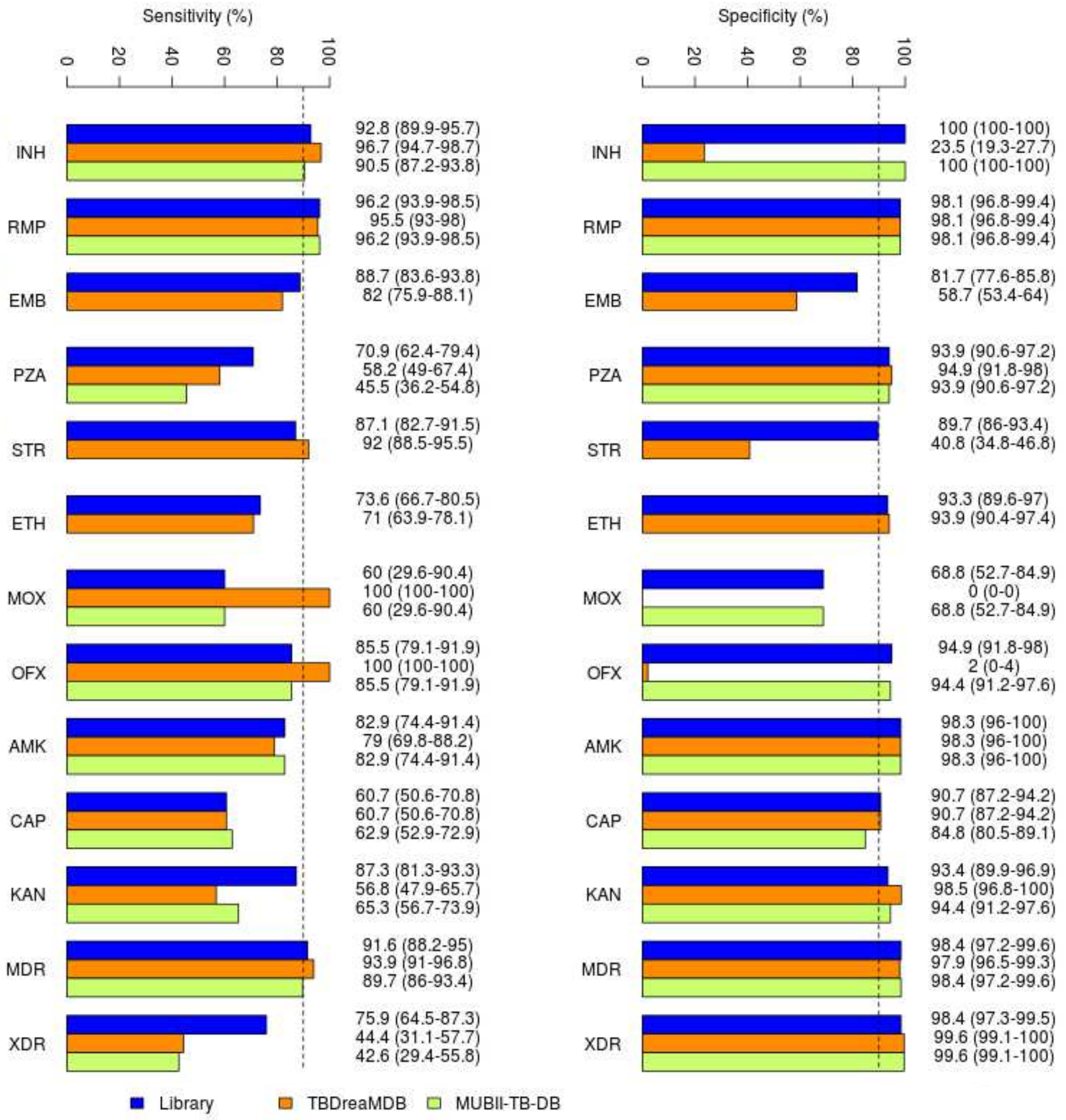

Inferred analytical accuracies (sensitivity and specificity) of the curated drug resistance mutation library versus alternative drug resistance mutation databases: *TBDreaMDB* (Sandgren et al. 2009) and *MUBII-TB-DB* (Flandrois et al. 2014). For each library *in silico* inferred resistance phenotypes were compared to reported phenotypes obtained from conventional drug susceptibility testing. 95% confidence intervals are shown in brackets.

Supplementary Figure 4   Diagnostic accuracy across populations

The sensitivity and specificity of drug resistance mutations in the curated list is calculated for each drug, both within each population and overall, assuming the phenotypic drug susceptibility test is the reference standard. The point estimates are represented by solid rectangles with size proportional to the population size, where horizontal lines represent the 95% confidence intervals. The overall estimate is represented by a diamond with width representing the 95% confidence interval. Dotted vertical lines are drawn at the overall estimates. The data presented in this supplementary figure correspond to that of Table 2 in the main manuscript.

a) Isoniazid

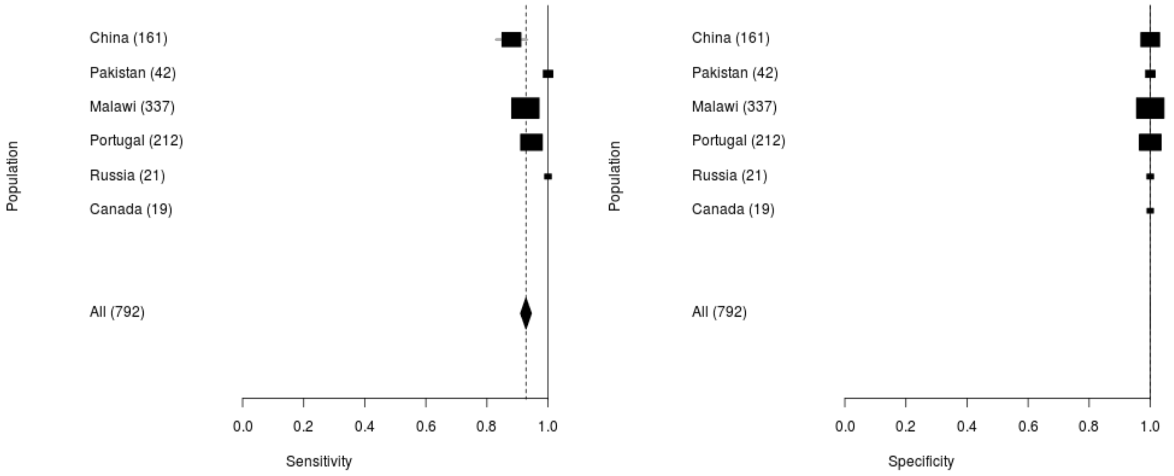

b) Rifampicin

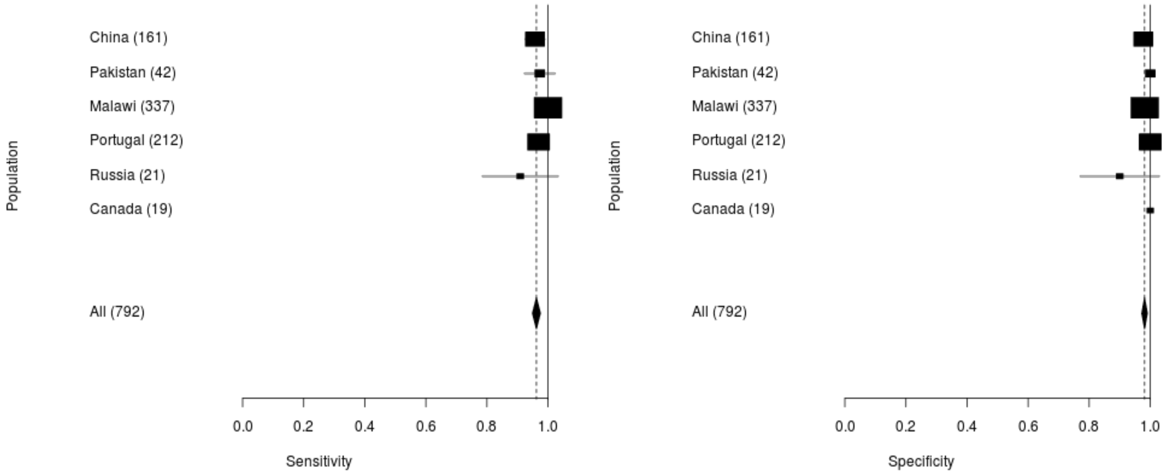

c) Ethambutol

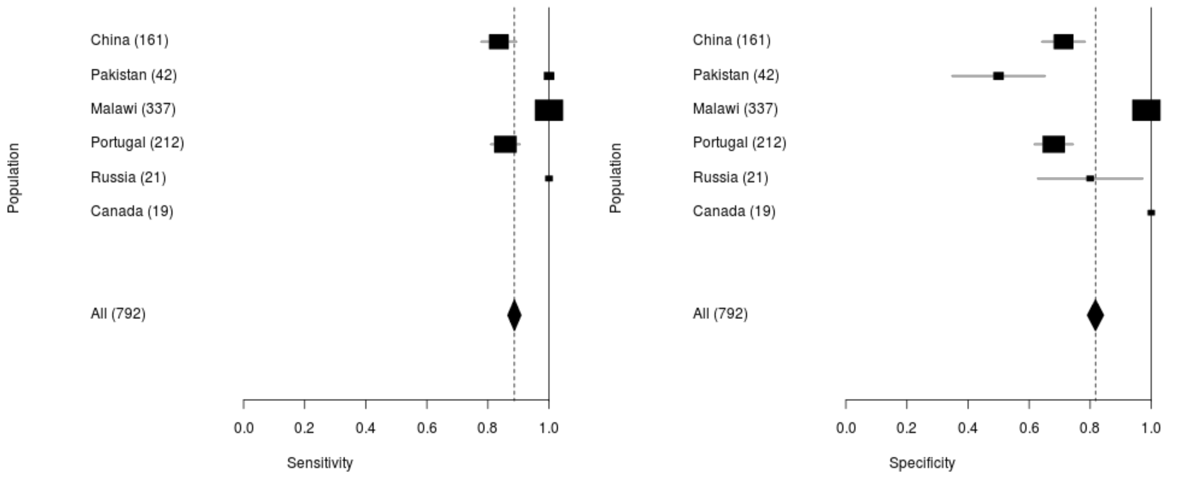

d) Pyrazinamide

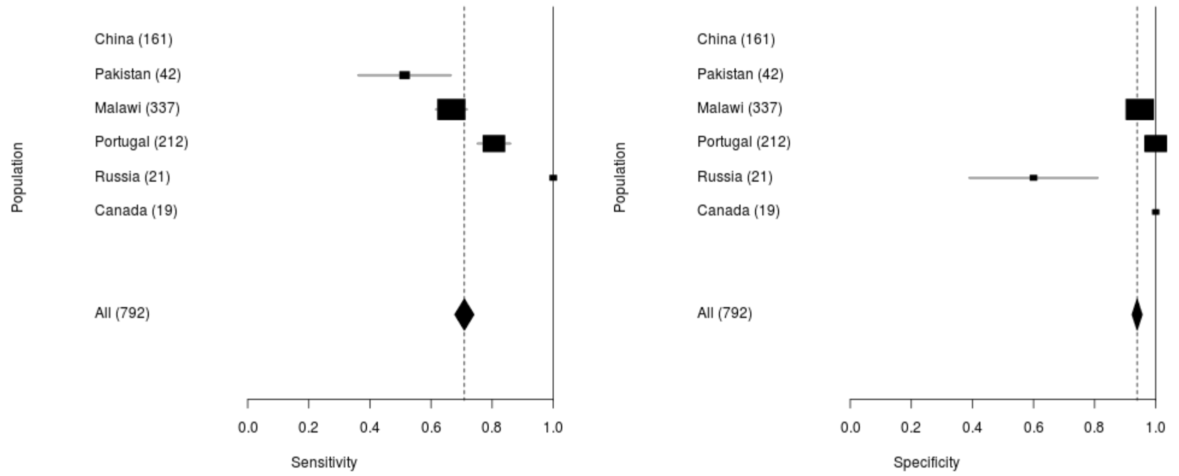

e) Streptomycin

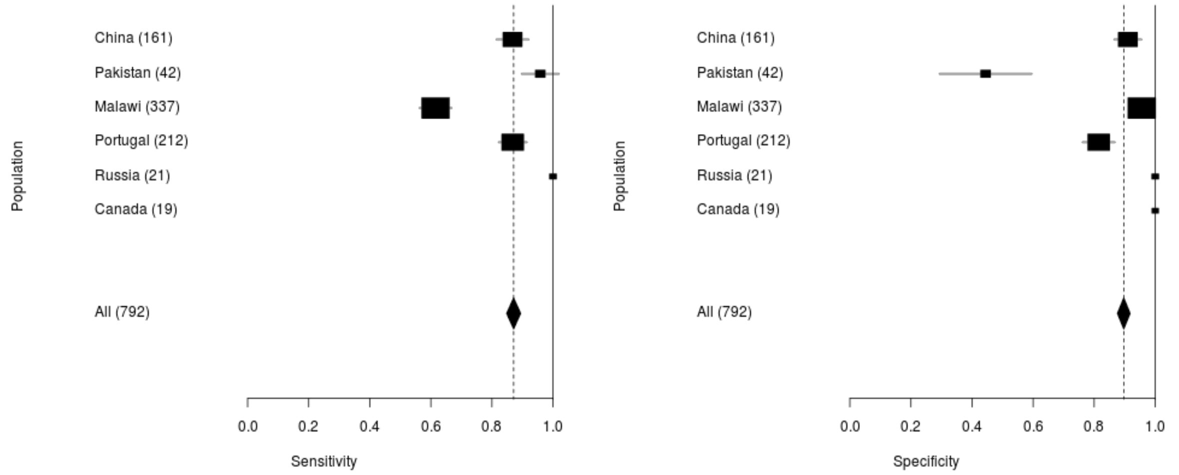

f) Ethionamide

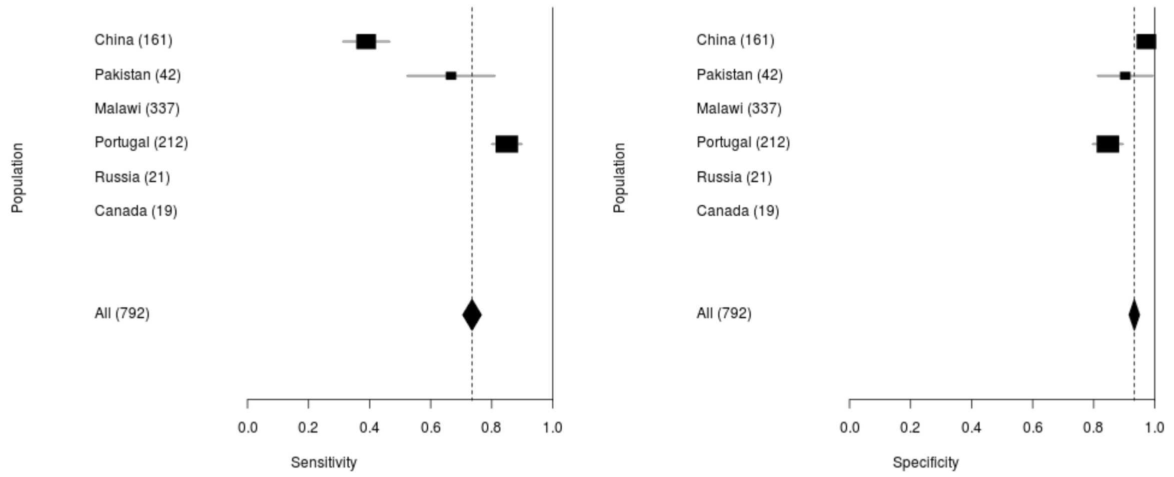

g) Moxifloxacin

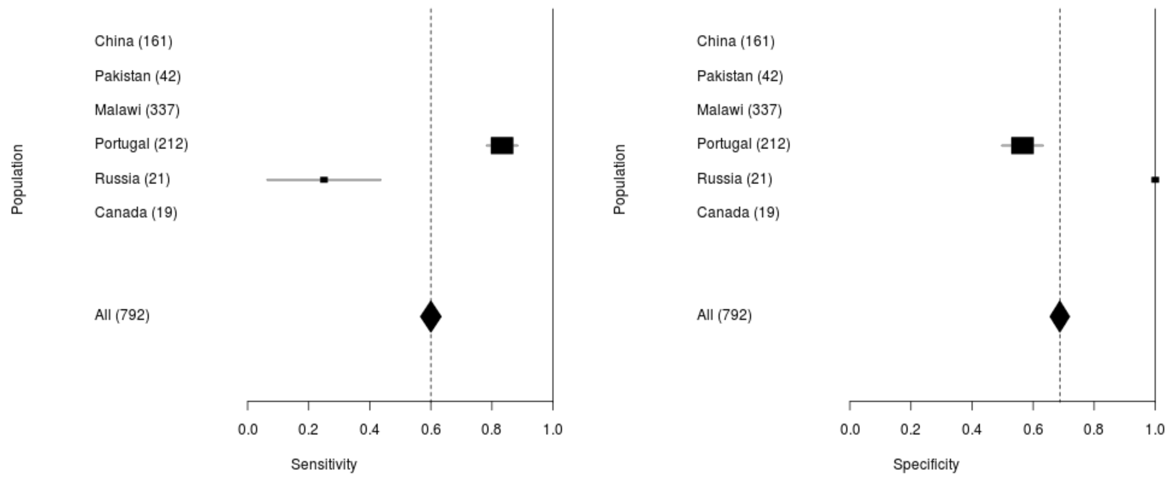

h) Ofloxacin

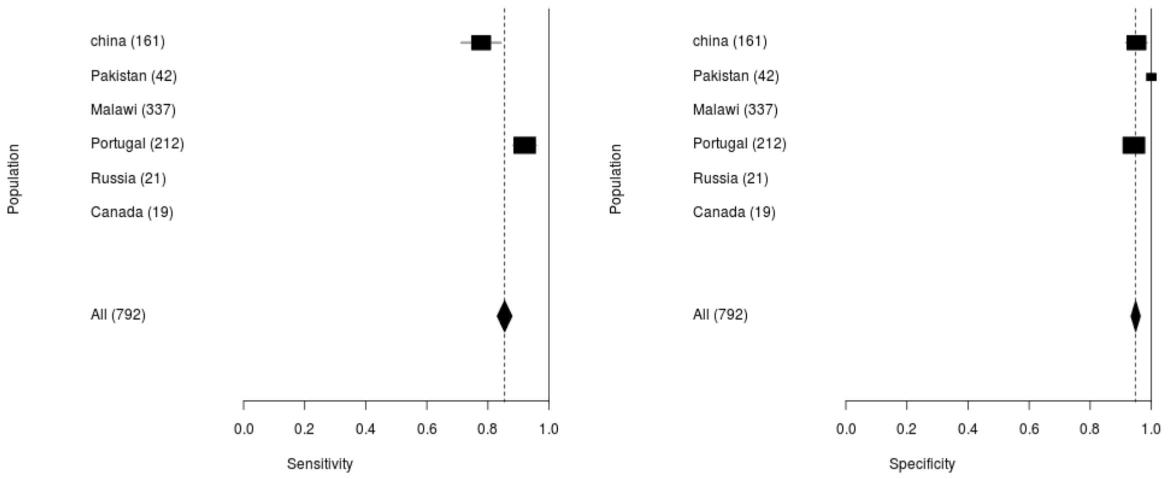

i) Amikacin

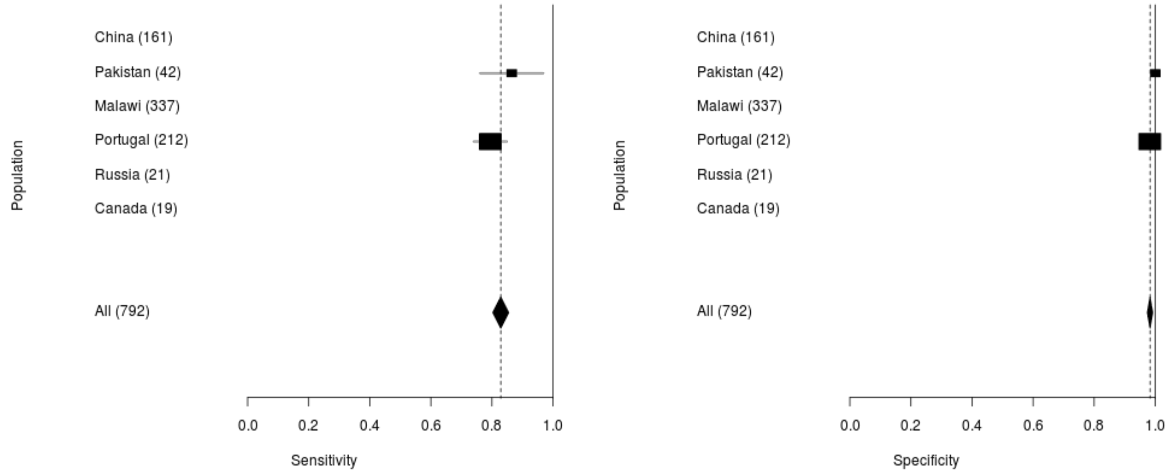

j) Capreomycin

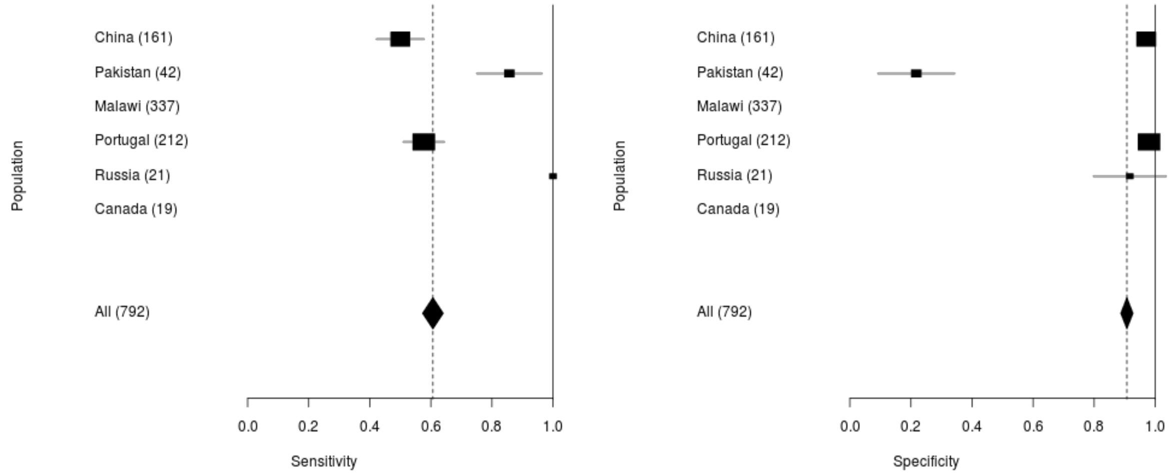

k) Kanamycin

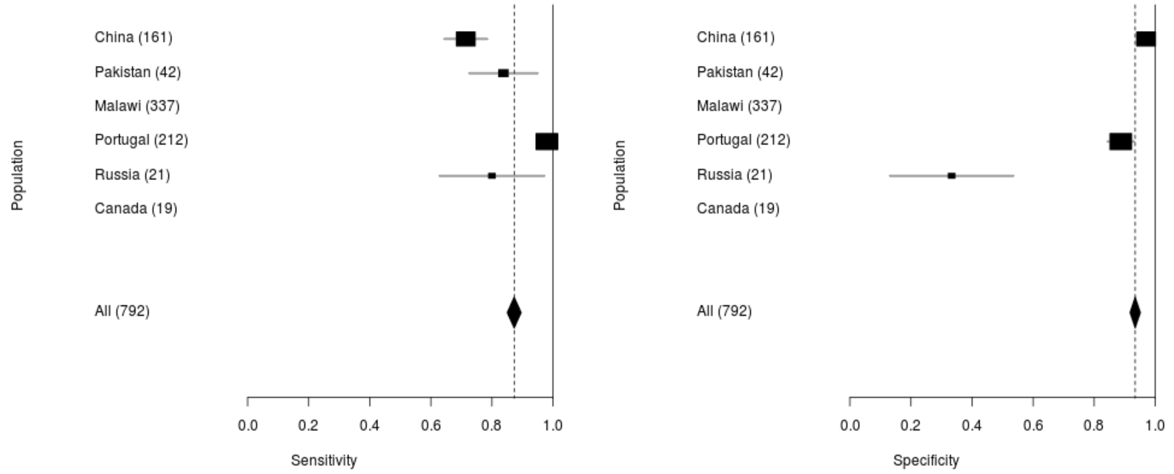

l) MDR

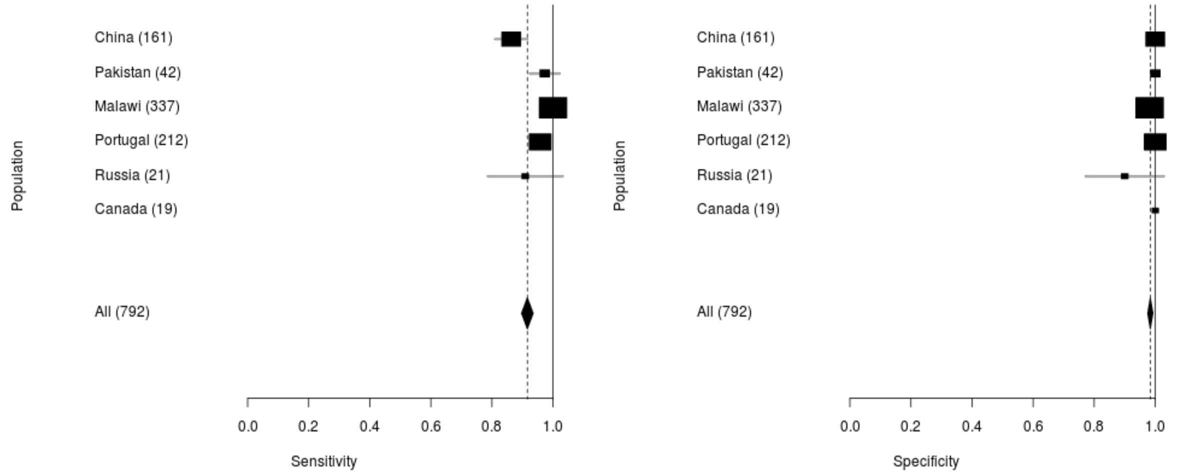

m) XDR

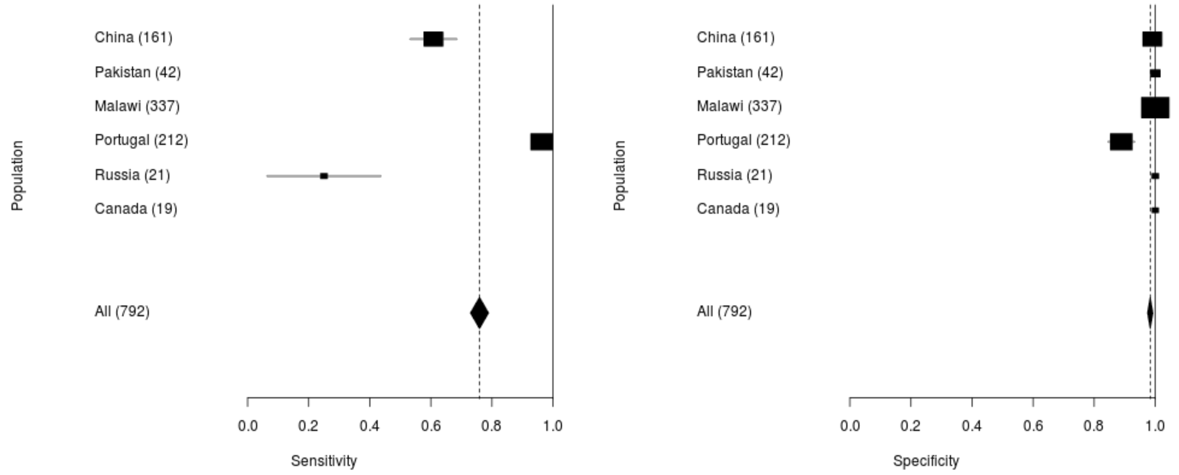

SUPPLEMENTARY REFERENCES

Ajbani, K., C. Nikam, M. Kazi, C. Gray, C. Boehme, K. Balan, A. Shetty, and C. Rodrigues. 2012. “Evaluation of Genotype MTBDRsl Assay to Detect Drug Resistance Associated with Fluoroquinolones, Aminoglycosides and Ethambutol on Clinical Sediments.” *PloS one* 7(11):e49433.

Ali, A., Z. Hasan, R. McNerney, K. Mallard, G. Hill-Cawthorne, F. Coll, M. Nair, A. Pain, T. G. Clark, and R. Hasan. 2015. “Whole Genome Sequencing Based Characterization of Extensively Drug-Resistant Mycobacterium Tuberculosis Isolates from Pakistan.” *PloS one* 10(2):e0117771.

Ando, H., S. Mitarai, Y. Kondo, T. Suetake, J.-I. Sekiguchi, S. Kato, T. Mori, and T. Kirikae. 2010. "Pyrazinamide Resistance in Multidrug-Resistant Mycobacterium Tuberculosis Isolates in Japan." *Clinical microbiology and infection : the official publication of the European Society of Clinical Microbiology and Infectious Diseases* 16(8):1164–68.

Andries, K., C. Villellas, N. Coeck, K. Thys, T. Gevers, L. Vranckx, N. Lounis, B. C. de Jong, and A. Koul. 2014. "Acquired Resistance of Mycobacterium Tuberculosis to Bedaquiline." *PloS one* 9(7):e102135.

Beckert, P., D. Hillemann, T. a. Kohl, J. Kalinowski, E. Richter, S. Niemann, and S. Feuerriegel. 2012. "rplC T460C Identified as a Dominant Mutation in Linezolid-Resistant Mycobacterium Tuberculosis Strains." *Antimicrobial Agents and Chemotherapy* 56:2743–45.

Boonaiam, S., a Chaiprasert, T. Prammananan, and M. Leechawengwongs. 2010. "Genotypic Analysis of Genes Associated with Isoniazid and Ethionamide Resistance in MDR-TB Isolates from Thailand." *Clinical microbiology and infection : the official publication of the European Society of Clinical Microbiology and Infectious Diseases* 16(4):396–99.

Brossier, F., N. Veziris, C. Truffot-Pernot, V. Jarlier, and W. Sougakoff. 2011. "Molecular Investigation of Resistance to the Antituberculous Drug Ethionamide in Multidrug-Resistant Clinical Isolates of Mycobacterium Tuberculosis." *Antimicrobial agents and chemotherapy* 55(1):355–60.

Casali, N. and V. Nikolayevskyy. 2012. "Microevolution of Extensively Drug-Resistant Tuberculosis in Russia." *Genome Res.* 22(4):735–45.

Coll, F., R. McNerney, J. A. Guerra-Assunção, J. R. Glynn, J. Perdigão, M. Viveiros, I. Portugal, A. Pain, N. Martin, and T. G. Clark. 2014. "A Robust SNP Barcode for Typing Mycobacterium Tuberculosis Complex Strains." *Nature Communications* 5:4812.

DeBarber, a E., K. Mdluli, M. Bosman, L. G. Bekker, and C. E. Barry. 2000. "Ethionamide Activation and Sensitivity in Multidrug-Resistant Mycobacterium Tuberculosis." *Proceedings of the National Academy of Sciences of the United States of America* 97(17):9677–82.

Engström, A., N. Morcillo, B. Imperiale, S. E. Hoffner, and P. Juréen. 2012. "Detection of First- and Second-Line Drug Resistance in Mycobacterium Tuberculosis Clinical Isolates by Pyrosequencing." *Journal of clinical microbiology* 50(6):2026–33.

Feuerriegel, S., C. U. Köser, and S. Niemann. 2014. "Phylogenetic Polymorphisms in Antibiotic Resistance Genes of the Mycobacterium Tuberculosis Complex." *The Journal of antimicrobial chemotherapy*.

Flandrois, J.-P., G. Lina, and O. Dumitrescu. 2014. "MUBII-TB-DB: A Database of Mutations Associated with Antibiotic Resistance in Mycobacterium Tuberculosis." *BMC bioinformatics* 15(1):107.

Gardy, J. L. et al. 2011. "Whole-Genome Sequencing and Social-Network Analysis of a Tuberculosis Outbreak." *N. Engl. J. Med.* 364(8):730–39.

Georghiou, S. B., M. Magana, R. S. Garfein, D. G. Catanzaro, A. Catanzaro, and T. C. Rodwell. 2012. "Evaluation of Genetic Mutations Associated with Mycobacterium Tuberculosis Resistance to Amikacin, Kanamycin and Capreomycin: A Systematic Review." *PloS one* 7(3):e33275.

Guerra-Assunção, J., A. Crampin, R. Houben, T. Mzembe, K. Mallard, F. Coll, P. Khan, L. Banda, A. Chiwaya, R. Pereira, R. McNerney, P. Fine, J. Parkhill, T. Clark, and J. Glynn. 2015. "Large-Scale Whole Genome Sequencing of M. Tuberculosis Provides Insights into Transmission in a High Prevalence Area." *eLife* 4:e05166.

Hartkoorn, R. C., S. Uplekar, and S. T. Cole. 2014. "Cross-Resistance between Clofazimine and Bedaquiline through Upregulation of MmpL5 in Mycobacterium Tuberculosis." *Antimicrobial agents and chemotherapy* 58(5):2979–81.

Helb, D. et al. 2010. "Rapid Detection of Mycobacterium Tuberculosis and Rifampin Resistance by Use of on-Demand, near-Patient Technology." *Journal of clinical microbiology* 48(1):229–37.

Hillemann, D., S. Rüsch-Gerdes, and E. Richter. 2008. "In Vitro-Selected Linezolid-Resistant Mycobacterium Tuberculosis Mutants." *Antimicrobial Agents and Chemotherapy* 52(2):800–801.

Jin, J., Y. Zhang, X. Fan, N. Diao, L. Shao, F. Wang, P. Hu, S. Wang, X. Weng, and W. Zhang. 2012. "Evaluation of the GenoType® MTBDRplus Assay and Identification of a Rare Mutation for Improving MDR-TB Detection." *The international journal of tuberculosis and lung disease : the official journal of the International Union against Tuberculosis and Lung Disease* 16(4):521–26.

Jnawali, H. N., S. C. Hwang, Y. K. Park, H. Kim, Y. S. Lee, G. T. Chung, K. H. Choe, and S. Ryoo. 2013. "Characterization of Mutations in Multi- and Extensive Drug Resistance among Strains of Mycobacterium Tuberculosis Clinical Isolates in Republic of Korea." *Diagnostic microbiology and infectious disease* 76(2):187–96.

Liu, Q., T. Luo, J. Li, J. Mei, and Q. Gao. 2013. "Triplex Real-Time PCR Melting Curve Analysis for Detecting Mycobacterium Tuberculosis Mutations Associated with Resistance to Second-Line Drugs in a Single Reaction." *The Journal of antimicrobial chemotherapy* 68(5):1097–1103.

Maruri, F., T. R. Sterling, A. W. Kaiga, A. Blackman, Y. F. van der Heijden, C. Mayer, E. Cambau, and A. Aubry. 2012. "A Systematic Review of Gyrase Mutations Associated with Fluoroquinolone-Resistant Mycobacterium Tuberculosis and a Proposed

Gyrase Numbering System.” *The Journal of antimicrobial chemotherapy* 67(4):819–31.

Morlock, G. and B. Metchock. 2003. “ethA, inhA, and katG Loci of Ethionamide-Resistant Clinical Mycobacterium Tuberculosis Isolates.” *Antimicrobial agents …* 47(12):3799–3805.

Moure, R., G. Tudó, R. Medina, E. Vicente, J. M. Caldito, M. G. Codina, P. Coll, M. Español, J. Gonzalez-Martin, E. Rey-Jurado, M. Salvadó, M. T. Tórtola, and F. Alcaide. 2013. “Detection of Streptomycin and Quinolone Resistance in Mycobacterium Tuberculosis by a Low-Density DNA Array.” *Tuberculosis (Edinburgh, Scotland)* 93(5):508–14.

Nebenzahl-Guimaraes, H., K. R. Jacobson, M. R. Farhat, and M. B. Murray. 2013. “Systematic Review of Allelic Exchange Experiments Aimed at Identifying Mutations That Confer Drug Resistance in Mycobacterium Tuberculosis.” *Journal of Antimicrobial Chemotherapy* 1–12.

Perdigão, J., H. Silva, D. Machado, R. Macedo, F. Maltez, C. Silva, L. Jordao, I. Couto, K. Mallard, F. Coll, G. A. Hill-Cawthorne, R. McNerney, A. Pain, T. G. Clark, M. Viveiros, and I. Portugal. 2014. “Unraveling Mycobacterium Tuberculosis Genomic Diversity and Evolution in Lisbon, Portugal, a Highly Drug Resistant Setting.” *BMC genomics* 15(1):991.

Safi, H., S. Lingaraju, A. Amin, S. Kim, M. Jones, M. Holmes, M. McNeil, S. N. Peterson, D. Chatterjee, R. Fleischmann, and D. Alland. 2013. “Evolution of High-Level Ethambutol-Resistant Tuberculosis through Interacting Mutations in Decaprenylphosphoryl-B-D-Arabinose Biosynthetic and Utilization Pathway Genes.” *Nature genetics* 45(10):1190–97.

Sandgren, A., M. Strong, P. Muthukrishnan, B. K. Weiner, G. M. Church, and M. B. Murray. 2009. “Tuberculosis Drug Resistance Mutation Database.” *PLoS Med.* 6(2):e1000002.

Sekiguchi, J.-I., T. Nakamura, T. Miyoshi-Akiyama, F. Kirikae, I. Kobayashi, E. Augustynowicz-Kopec, Z. Zwolska, K. Morita, T. Suetake, H. Yoshida, S. Kato, T. Mori, and T. Kirikae. 2007. “Development and Evaluation of a Line Probe Assay for Rapid Identification of pncA Mutations in Pyrazinamide-Resistant Mycobacterium Tuberculosis Strains.” *Journal of clinical microbiology* 45(9):2802–7.

Shi, W., J. Chen, J. Feng, P. Cui, S. Zhang, X. Weng, W. Zhang, and Y. Zhang. 2014. “Aspartate Decarboxylase (PanD) as a New Target of Pyrazinamide in Mycobacterium Tuberculosis.” *Emerging Microbes & Infections* 3(000):e58.

Shi, X., C. Zhang, M. Shi, M. Yang, Y. Zhang, J. Wang, H. Shen, G. Zhao, and X. Ma. 2013. “Development of a Single Multiplex Amplification Refractory Mutation System PCR for the Detection of Rifampin-Resistant Mycobacterium Tuberculosis.” *Gene* 530(1):95–99.

Slayden, R. a and C. E. Barry. 2000. “The Genetics and Biochemistry of Isoniazid Resistance in Mycobacterium Tuberculosis.” *Microbes and infection / Institut Pasteur* 2(6):659–69.

Stoffels, K., V. Mathys, M. Fauville-Dufaux, R. Wintjens, and P. Bifani. 2012. “Systematic Analysis of Pyrazinamide-Resistant Spontaneous Mutants and Clinical Isolates of Mycobacterium Tuberculosis.” *Antimicrobial agents and chemotherapy* 56(10):5186–93.

Tan, Y., Z. Hu, T. Zhang, X. Cai, H. Kuang, Y. Liu, J. Chen, F. Yang, K. Zhang, S. Tan, and Y. Zhao. 2013. “Role of pncA and rpsA Gene Sequencing in Diagnosis of Pyrazinamide Resistance in Mycobacterium Tuberculosis Isolates from Southern China.” *Journal of clinical microbiology* (October).

Wang, X., J. Jiao, W. Xu, X. Chai, Z. Li, and Q. Wang. 2013. “A Simple, Rapid and Economic Method for Detecting Multidrug-Resistant Tuberculosis.” *The Brazilian journal of infectious diseases : an official publication of the Brazilian Society of Infectious Diseases* (x x):2–6.

Zhang, H. et al. 2013. “Genome Sequencing of 161 Mycobacterium Tuberculosis Isolates from China Identifies Genes and Intergenic Regions Associated with Drug Resistance.” *Nature genetics* 45(10):1255–60.

Zhang, S., J. Chen, W. Shi, W. Liu, W. Zhang, and Y. Zhang. 2013. “Mutations in panD Encoding Aspartate Decarboxylase Are Associated with Pyrazinamide Resistance in Mycobacterium Tuberculosis.” *Emerging Microbes & Infections* 2(6):e34.

Zhang, X., L. Liu, Y. Zhang, G. Dai, H. Huang, and Q. Jin. 2014. “Genetic Determinants Involved in P-Aminosalicylic Acid Resistance in Clinical Isolates from Tuberculosis Patients in North of China during 2006-2012.” *Antimicrobial agents and chemotherapy* (November).

Zhao, F., X.-D. Wang, L. N. Erber, M. Luo, A. Guo, S. Yang, J. Gu, B. J. Turman, Y. Gao, D. Li, Z. Cui, Z. Zhang, L. Bi, A. D. Baughn, X.-E. Zhang, and J.-Y. Deng. 2014. “Binding Pocket Alterations in Dihydrofolate Synthase Confer Resistance to Para-Aminosalicylic Acid in Clinical Isolates of Mycobacterium Tuberculosis.” *Antimicrobial agents and chemotherapy* 58(3):1479–87.

Zimenkov, D. V, O. V Antonova, A. V Kuz’min, Y. D. Isaeva, L. Y. Krylova, S. a Popov, A. S. Zasedatelev, V. M. Mikhailovich, and D. a Gryadunov. 2013. “Detection of Second-Line Drug Resistance in Mycobacterium Tuberculosis Using Oligonucleotide Microarrays.” *BMC infectious diseases* 13(1):240.
